# Voxel segmentation-based 3D building detection algorithm for airborne LIDAR data

**DOI:** 10.1371/journal.pone.0208996

**Published:** 2018-12-28

**Authors:** Liying Wang, Yan Xu, Yu Li, Yuanding Zhao

**Affiliations:** School of Geomatics, Liaoning Technical University, Fuxin, Liaoning, China; University of Pittsburgh, UNITED STATES

## Abstract

Among traditional Light Detection And Ranging (LIDAR) data representations such as raster grid, triangulated irregular network, point clouds and octree, the explicit 3D nature of voxel-based representation makes it a promising alternative. Despite the benefit of voxel-based representation, voxel-based algorithms have rarely been used for building detection. In this paper, a voxel segmentation-based 3D building detection algorithm is developed for separating building and nonbuilding voxels. The proposed algorithm first voxelizes the LIDAR point cloud into a grayscale voxel structure in which the grayscale of the voxel corresponds to the quantized mean intensity of the LIDAR points within the voxel. The voxelized dataset is segmented into multiple 3D-connected regions depending on the connectivity and grayscale similarity among voxels. The 3D-connected regions corresponding to the building roof and facade are detected sequentially according to characteristics such as their area, density, elevation difference and location. The obtained results for the detected buildings are evaluated by the LIDAR data provided by working group III/4 of ISPRS, which demonstrate a high rate of success. Average completeness, correctness, quality, and kappa coefficient indexes values of 90.0%, 96.0%, 88.1% and 88.7%, respectively, are obtained for buildings.

## Introduction

As buildings are indispensable components of 3D geographic information productions, studies on automatic, high-precision and rapid building detection and reconstruction attract wide attention. Airborne Light Detection And Ranging (LIDAR) data, which can provide dense, accurate, and georeferenced true 3D point clouds and the intensity of the returned signal, appear to be an ideal data source for detecting 3D buildings.

Building detection methods for building detection from airborne LIDAR data generelly separate points on buildings from those on the surfaces of other landscape content such as ground, trees and roads. The classicmethods can be grouped into four categories. The first is based on a filtering procedure that first classifies the LIDAR points into ground and nonground points using an iterative calculation based on a Triangulated Irregular Network (TIN) structure or certain operators designed based on mathematical morphology, terrain slope, or local elevation difference to compute a Digital Terrain Model (DTM). A normalized Digital Surface Model (nDSM) is generated by subtracting the DTM from the DSM. Image segmentation techniques are used to detect building regions within the nDSM [1–7]. The second category of methods is concerned with growing homogeneous regions to identify seed points located on planar surface patches and then enlarge surface patches around the seed points using smoothness constraints or other similarity criteria [8–11]. The third category is segmentation-based methods, which segment LIDAR points into individual independent processing units using local surface properties as a similarity criterion and detect building units using building characteristics [12–21]. The last category is clustering methods, which associate each LIDAR point with a feature vector that consists of geometric and/or radiometric measures and segment LIDAR points in feature spaces using a clustering technique such as *k*-means, maximum likelihood and fuzzy-clustering [22–32]. The abovementioned methods can be used on TINs, raster grids, point clouds or octrees, but all of them have limitations. TINs and raster grids, which assign only one distinct elevation value in relation to the same horizontal coordinate, simplify the 3D content of LIDAR data to 2.5D and cause the loss of interior returns information. This can affect the integrity of raster grid- and TIN-based building detection methods. Point clouds, the original expression of LIDAR data, can completely retain the 3D information of the raw data but cannot explicitly represent spatial structure and topological information. This leads to difficulties in the design of building detection methods based on point clouds. An octree structure recursively subdivides the 3D space of LIDAR data into eight subspaces (nodes) until each contains no point or points fewer than a predefined number of points, or until reaching a predefined subdivision depth or a minimal voxel size [33]. The node size may not be optimal in terms of representation of the LIDAR data. Since nodes of an octree have different sizes, but adjacency relationships among nodes are difficult to model. This also increases difficulties in the design of building detection methods based on octrees. To overcome the restrictions for TIN-, raster grid-, point cloud- and octree-based methods, a Voxel Segmentation-based Building Detection (VSBD) algorithm is proposed. The proposed VSBD algorithm regularizes the LIDAR data into a Grayscale Voxel Structure (GVS), in which the grayscale voxel corresponds to the quantized mean intensity of the LIDAR points within the voxel. The GVS is segmented into multiple 3D-connected regions depent on connectivity and grayscale similarity among voxels. Finally, the 3D-connected regions corresponding to the building roof and facade are detected sequentially according to their characteristics. The GVS model adopted in the proposed VSBD algorithm has obvious advantages. First, it is a 3D structure and can represent multiple returns of LIDAR data simultaneously, facilitating more comprehensive utilization of multiple returns information. Second, it explicitly represents topological and spatial structure information, facilitating the design of building detection algorithms. Third, voxels in GVS have a fixed size, and a voxel’s nearest neighbor can be found by searching its spatial neighbor voxels. This is more flexible than the octree structure.It fuses elevation and intensity information simultaneously, supporting building detection in areas where buildings are next to nonbuilding objects but with different intensities. Despite the advantages of GVS, GVS-based algorithms have been rarely used for 3D building detection. The existing voxel-based algorithms are designed based on binary (a voxel with value 0 or 1) or density (a voxel has a value corresponding to the number of LIDAR points within the voxel) voxel representation and have been used in applications such as spatial indexes [34–35], forest structure [36–38], biomass [39], and topographic and geographic representations [40–41]. The advantages of the proposed VSBD algorithm are that it is designed based on a GVS and, as a 3D building detection algorithm, it makes better use of 3D connectivity among voxels and intensity information. Its building detection results can also be used to create a 3D model of buildings.

The goal of this paper is to develop a novel voxel segmentation based algorithm to precisely detect buildings from the constructed GVS model. The organization of the paper is as follows. The LIDAR data used in the test and the proposed VSBD algorithm are described in the “Data and methods” Section. The results of the experiments are shown and discussed in the“Results” Section. Finally, an outlook and a summary are presented in the “Discussion and conclusions” Section.

## Data and methods

### Test data

**Fig 1:**
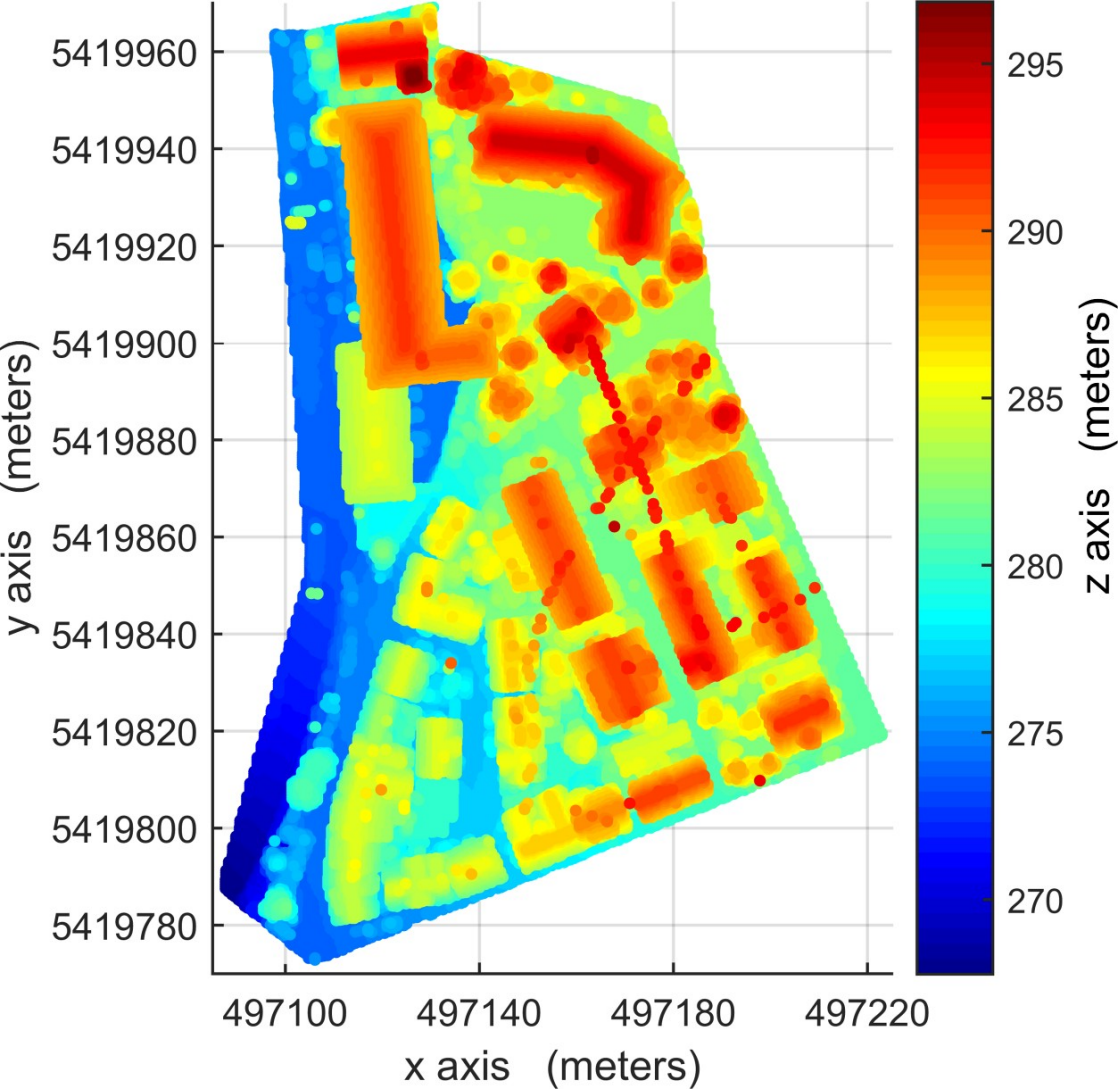
LIDAR point cloud data of Area 1, absolute elevations of 265-297m.

**Fig 2:**
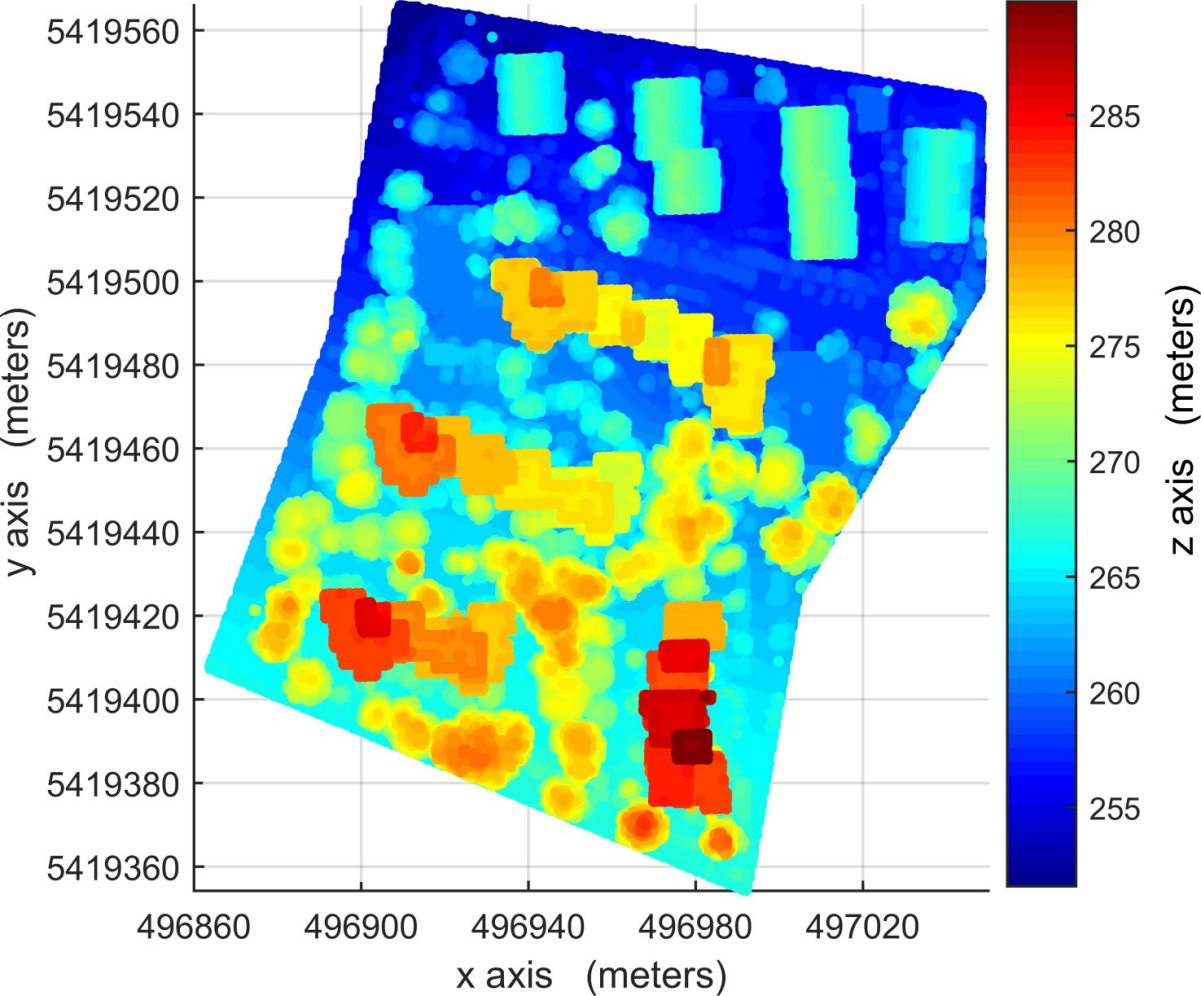
LIDAR point cloud data of Area 2, absolute elevations of 250-290m.

**Fig 3:**
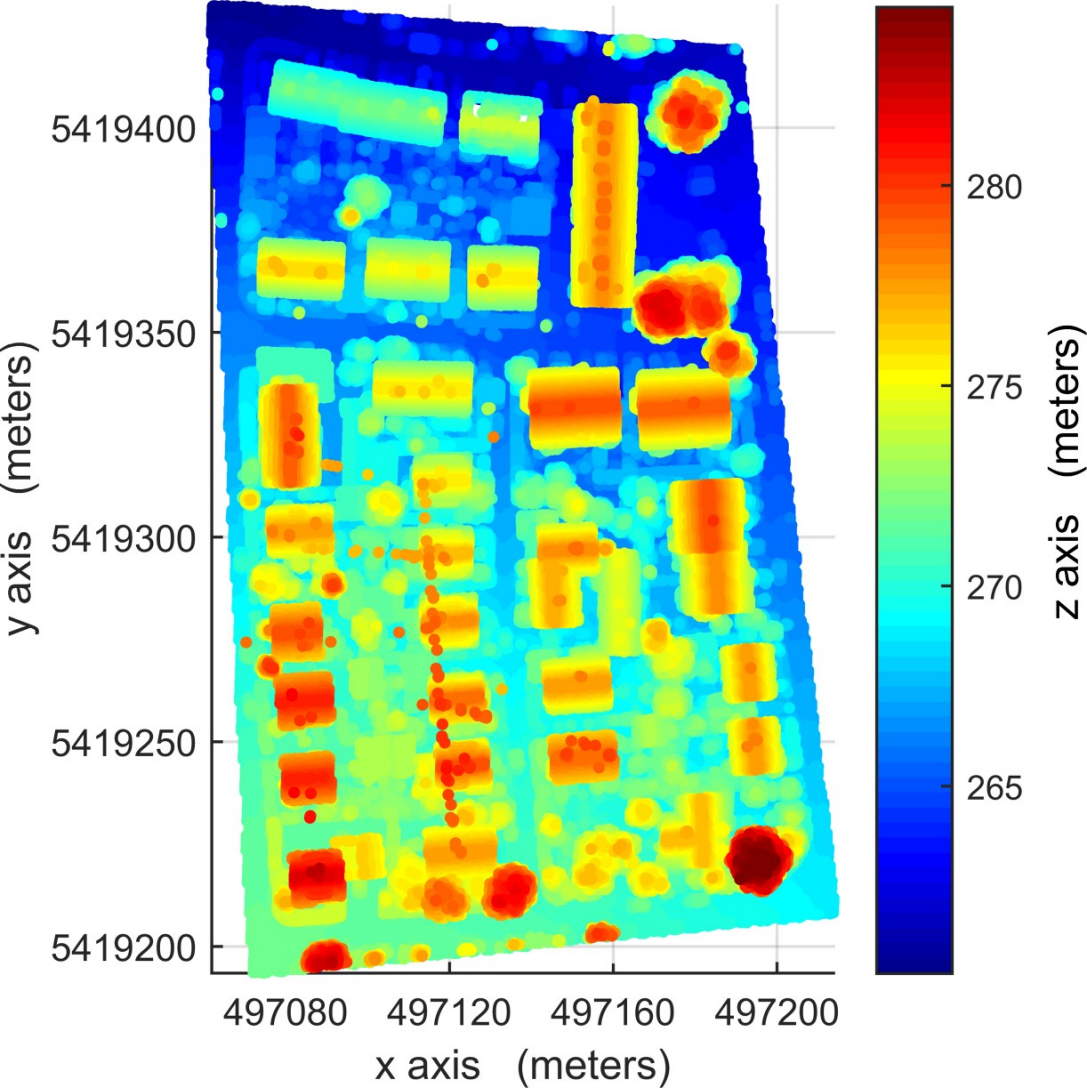
LIDAR point cloud data of Area 1, absolute elevations of 265-297m.

The LIDAR data used in this paper were provided by Working Group (WG) III/4 of ISPRS from the Vaihingen area of Germany in the context of the ‘ISPRS test project on urban classification and 3D building reconstruction’. The data [42] must be requested via the link http://www2.isprs.org/commissions/comm3/wg4/data-request-form2.html. Other conditions on the use of the data include that a specified paper [42] must be cited and an acknowledgment must be included. The data consist of three testing sites, Areas 1, 2 and 3, see Figs 1, 2 and 3, respectively. The dataset was captured on August 21, 2008 by a Leica ALS50 system with a 45° field of view and a mean flying height of 500 m above ground. In an area covered by one strip the mean point density is 4 points / m^2^. Multiple returns and their intensities were recorded. Three testing sites are representatives of building areas of diverse types and were used for the quantitative analysis. Area 1 (37 buildings and 105 trees) is characterized by dense development consisting of historic buildings with rather complex shapes along with roads and trees. Area 2 (14 buildings and 162 trees) is characterized by a few high-rising residential buildings surrounded by trees. Area 3 (56 buildings and 155 trees) is a residential area with detached houses and many surrounding trees. The building-truth data of Area 1 was automatically created using commercial software. The building-truth data of Area 2 and 3 were prepared by ISPRS-WGIII/4. They were used to evaluate the accuracy of the proposed VSBD algorithm quantitatively.

### Methods

The proposed VSBD algorithm comprises three steps: voxelization of the LIDAR data, segmentation of the voxelized dataset, and detection of the building roofs and facades. In the first step, the LIDAR point cloud is voxelized into a GVS model to reconstruct the LIDAR data, in which the voxel grayscale corresponds to the quantized mean intensity of the LIDAR points within the voxel. In the next step, the voxelized dataset is segmented into multiple 3D-connected regions depending on the connectivity and grayscale similarity among voxels. In the last step, the 3D-connected regions corresponding to the building roof and facade are detected sequentially using their characteristics.

#### Voxelization of LIDAR data

Voxelization of LIDAR data can be performed by dividing the entire scene volume into a collection of 3D regular cubes (called voxels), in each of which the LIDAR points to 3D voxels are allocated and voxel values are assigned according to the attribute values of the LIDAR point(s) within the corresponding voxels.

For a given LIDAR point cloud, *P* = {*p*_*i*_ (*x*_*i*_, *y*_*i*_, *z*_*i*_), *i* = 1, …, *n*}, where *i* is the index of LIDAR points, *n* is the number of LIDAR points, *p*_*i*_ represents the *i*th LIDAR point, and (*x*_*i*_, *y*_*i*_, *z*_*i*_) represents the point coordinates of *i*th point along *x*, *y* and *z* axes, respectively. An Axis-Aligned Bounding Box (AABB) is used to determine the scene volume or the extent of *P*. The AABB = {(*x*, *y*, *z*)|*x*_*min*_ ≤ *x* ≤ *x*_*max*_, *y*_*min*_ ≤ *y* ≤ *y*_*max*_, *z*_*min*_ ≤ *z* ≤ *z*_*max*_}, where (*x*_*max*_, *y*_*max*_, *z*_*max*_) and (*x*_*min*_, *y*_*min*_, *z*_*min*_) are the maximum and minimum, respectively, of *x*, *y* and *z*-coordinates in *P*, *x*_*max*_ (*y*_*max*_, *z*_*max*_) = max {*x*_*i*_ (*y*_*i*_, *z*_*i*_), *i* = 1, …, *n*}, *x*_*min*_ (*y*_*min*_, *z*_*min*_) = min {*x*_*i*_ (*y*_*i*_, *z*_*i*_), *i* = 1, …, *n*}.

The AABB can be divided into uniform 3D voxels according to the voxel resolution. The voxel resolution is the most important parameter during the voxelization of a given LIDAR point cloud. If the resolution is too high, the number of voxels that contain LIDAR points does not appear to change while the number of voxels that contain no LIDAR point becomes large. This results in increased redundancy. If the resolution is too low, more LIDAR points fall into a voxel, which increases the information loss since a voxel gets only one value. To minimize the redundancy and reduce the information loss, a suitable resolution must be used. In the case of an idealized sampling where the LIDAR points are evenly distributed and form a regularly spaced grid, the horizontal resolution can be determined based on the 2D point spacing of a given LIDAR point cloud using the optimal criterion, that is, whether a voxel contains only one LIDAR point [43], as follows. Δx=Δy=Axyn, where Δ*x* and Δ*y* are the voxel resolutions along the *x* and *y* axes, respectively, and *A*_*xy*_ is the horizontal projected area of LIDAR points. Setting the vertical resolution (Δ*z*) has two typical schemes [40, 43]. In the first,

Δz=min[Axzn,Ayzn], (1)

*A*_*xz*_ (*A*_*yz*_) is the projected area of LIDAR points in the *x*-*z* (*y*-*z*) plane [40]; in the second, Δ*z* = Δ*x* [43]. The former is suitable for representing the raw LIDAR point cloud and ground filtering, and the latter is a more appropriate scheme for building detection. This is elaborated on the“Experimental results and discussion” section.

Depending on the voxel resolution, the AABB is divided into rows, columns and layers, and a 3D array is established; the set of voxels for the constructed 3D array is denoted as *V* = {*v*_*j*_ (*r*_*j*_, *c*_*j*_, *l*_*j*_), *j* = 1, …, *m*}, where *j* is the index of the voxels, *m* is the number of voxels, *v*_*j*_ is the voxel value of *j*th voxel (its value will be assigned later), and (*r*_*j*_, *c*_*j*_, *l*_*j*_) are the coordinates of *j*th voxel in the 3D array.

The LIDAR points are allocated to the voxels of *V* using the formula,

$$r_{i}=\left\lfloor \frac{x_{i}-x_{min}}{\Delta x}\right\rfloor ,c_{i}=\left\lfloor \frac{y_{i}-y_{min}}{\Delta y}\right\rfloor ,l_{i}=\left\lfloor \frac{z_{i}-z_{min}}{\Delta z}\right\rfloor .$$ (2)

The voxel value is assigned according to the intensity values of the LIDAR points within the corresponding voxels. The values of filled voxels are defined as the mean intensities of the LIDAR points, and the values of empty voxels are set to zero. The voxel values are quantized to {0, …, 255} levels. The generated 3D array with 256 gray levels is the constructed GVS and is used as the source data for the subsequent segmentation.

Moreover, there are typically outliers in LIDAR data, which originate from the multiple reflections of object structures such as trees, the uneven reflection characteristics of the objects themselves such as buildings, and the reflections of birds or suspended objects at higher altitudes. The accuracy and efficiency of the established GVS are greatly influenced by outliers. A histogram examination technique is used to avoid the effect of outliers. An elevation histogramthat reveals the overall distribution characteristics of elevation is generated. Elevation thresholds are determined by visual evaluation to eliminate the lowest and highest tails. LIDAR points that are higher or lower than the highest (*T*_*h*_) or lowest (*T*_*l*_) elevation thresholds are removed from the dataset.

## Segmentation of the voxelized dataset

The objective of segmentation is to spatially merge voxels with connectivity and similar grayscale properties into one 3D-connected region. Suppose that the constructed GVS, denoted by *V*, has a total of *k* connected regions in 3D space. The task of segmentation is to assign *k* labels to the voxels of *V* in a such way that all of the voxels in each 3D-connected region have the same label and voxels in different 3D-connected regions have different labels.

Based on the criterion that the voxels belong to one 3D-connected region if they are 3D-connected and have similar grayscales, a 3D-connected region labeling algorithm is proposed as follows. Iterate over the voxels of *V* until the *j*th voxel is found that has not yet been labeled. Suppose that *v*_*j*_ = *u* and labels *L*_1_, …, *L*_*d*−1_ (where *d* is the index of 3D-connected regions, 1 ≤ *d* ≤ *k*) have already been used. Choose a new label *L*_*d*_, and call the process LABEL(*j*, *u*, *L*_*d*_) which uses a depth-first strategy [44] to visit all the voxels in a 3D-connected region. After labeling the 3D-connected region that contains the *j*th voxel, continue to scan the voxels of *V* until all voxels have been labeled. Algorithm 1 shows the pseudo code of the LABEL(*j*, *u*, *L*_*d*_) process.

**Algorithm 1**: Pseudo code of the LABEL(*j*, *u*, *L*_*d*_) process

**Input**: *j* = the index of the voxel that has not yet been labeled, *u* = the voxel value of the *j*th voxel, *L*_*d*_ = the label of the *j*th voxel, and *V* = the set of voxels of the constructed 3D array

**Output**: voxels labeled with *L*_*d*_ belong to the *j*th voxel’s 3D-connected region

**1**    Label *j*th voxel with *L*_*d*_

**2**    Initialize a new stack to zero and store *j*th voxel into the stack

**3**    **if** the stack is empty, **then**

**4**            Stop the process

**5**    else

**6**            Pop top element *t*_*e*_ out of the stack

**7**            Label with *L*_*d*_ all unlabeled voxels in *V* adjacent to *t*_*e*_ and similar to *u* in grayscales (that is, their grayscales are within the statistical range of object corresponding to the *j*th voxel. The grayscale range of each object is determined later), and put these voxels into the stack

**8**            JUMP TO 3

Using different adjacency sizes (6-, 18- and 26-adjacency or others) in the process LABEL(*j*, *u*, *L*_*d*_) can obtain different segmentation results and affect the accuracy of building detection. The effects of adjacency size on the building detection results and the optimal adjacency size are studied in the “Experimental results and discussions” section.

**Fig 4:**
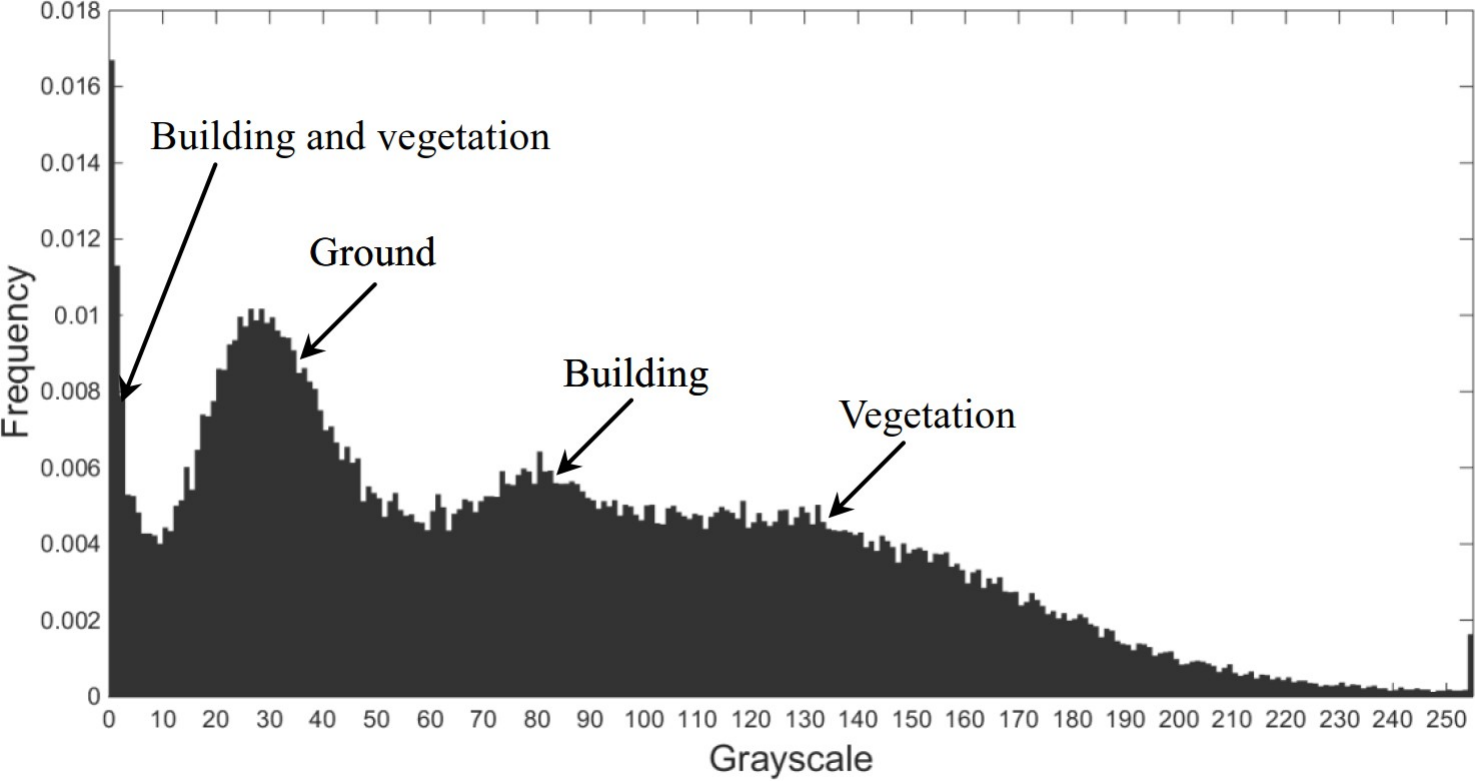
Nonzero value grayscale frequency histogram of testing site Area 3.

The grayscale range of each object in a given LIDAR point cloud is used as a similarity criterion. If the range is too large, voxels belonging to different objects may be grouped into one 3D-connected region and have the same label. If it is too small, voxels belonging to the same object may be segmented into multiple 3D-connected regions and have different labels. To determine the optimal value, a grayscale frequency histogram is calculated from all of the grayscales except 0 in *V*, as shown in Fig 4, which illustrates the example of testing site Area 3.

The grayscale distribution in Fig 4 exhibits multimodality. The multimodal distribution has four peaks (at approximately 1, 28, 80 and 133, respectively) and three valleys (at approximately 10, 61 and 103, denoted as *v*_1_, *v*_2_ and *v*_3_, respectively). Under the assumption that the multimodal distribution is a multimodal normal mixture distribution, the grayscale histogram can be characterized by a Gaussian Mixture Model (GMM) [45] with four Gaussian distributions as its components. Consequently, the mean and standard deviation of each Gaussian distribution can be determined, which are denoted as *μ*_*w*_ and *σ*_*w*_, respectively, where *w* is the index of Gaussian components, *w* = 1, 2, 3, 4. To ensure that the voxels belonging to the same Gaussian distribution are grouped into one 3D-connected region other than multiple 3D-connected regions, *μ*_*w*_, σ_*w*_, and the valleys are used to determine the range of each Gaussian distribution. For example, let *μ*_3_ *–m*_3*l*_ × σ_3_ *= v*_2_ and *μ*_3_ *+ m*_3*r*_ × σ_3_ *= v*_3_, then *m*_3*l*_ and *m*_3*r*_ can be determined, respectively. Consequently, [*μ*_3_ – *m*_3_*σ*_3_, *μ*_3_ + *m*_3_*σ*_3_] is the range of the third Gaussian distribution, where the multiplier *m*_3_ = max{*m*_3*l*_, *m*_3*r*_} is set according to the symmetry of the Gaussian distribution. The range of the other three Gaussian distributions can be determined in the same manner; notably, the minimum of the range for the first Gaussian distribution is set to zero but does not contain zero and the maximum of the range for the fourth Gaussian distribution is set to 255. Thus, the range of the four Gaussian distributions is determined and is denoted as [$r_{w}^{l},r_{w}^{r}$]. However, there may be overlap between adjacent distributions. To avoid distribution overlap and facilitate building detection, the ranges of the first and third Gaussian distributions use ($0,r_{1}^{r}$] and [$r_{3}^{l},r_{3}^{r}$], respectively, and the ranges of the second and fourth Gaussian distributions use ($r_{1}^{r},r_{3}^{l}$) and ($r_{3}^{r},r_{4}^{r}$], respectively. The ranges of Gaussian distributions are determined based on the first and third Gaussian distributions because they correspond to building object and the second and fourth distributions correspond to nonbuilding objects (the object(s) of each Gauss distribution that can be seen from the top view of the voxelized dataset and are used as prior knowledge). The grayscale ranges of objects in other testing sites can be determined in the same manner.

## Building roof and façade detection

Considering that an individual building is a 3D geometric shape, the grayscales of its voxels are similar, and the voxelized dataset obtained in the“Voxelisation of LIDAR point cloud” section is the 3D discretization of a variety of objects, it follows that voxels belonging to an individual building should form a 3D-connected region. The LIDAR points for a building rooftop are relatively complete and uniformly distributed because of fewer occlusions, whereas the LIDAR points corresponding to its façade are often incomplete or even missing and unevenly distributed because of trees or limitations of flight conditions. Consequently, the building roof and façade form separated 3D-connected regions. To ensure the integrity of building detection results, 3D-connected regions corresponding to the building roof are detected before the façade is detected.

**Fig 5:**
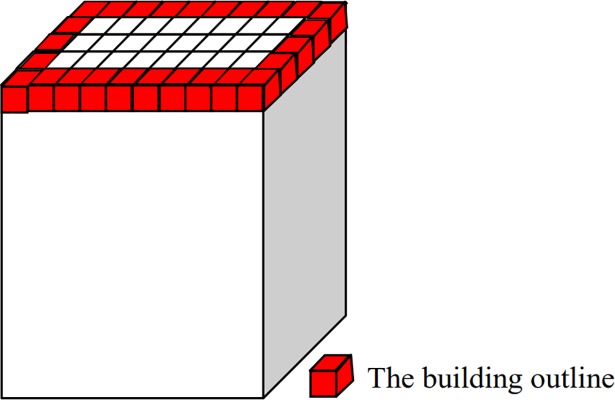
Contour line of a building.

**Fig 6:**
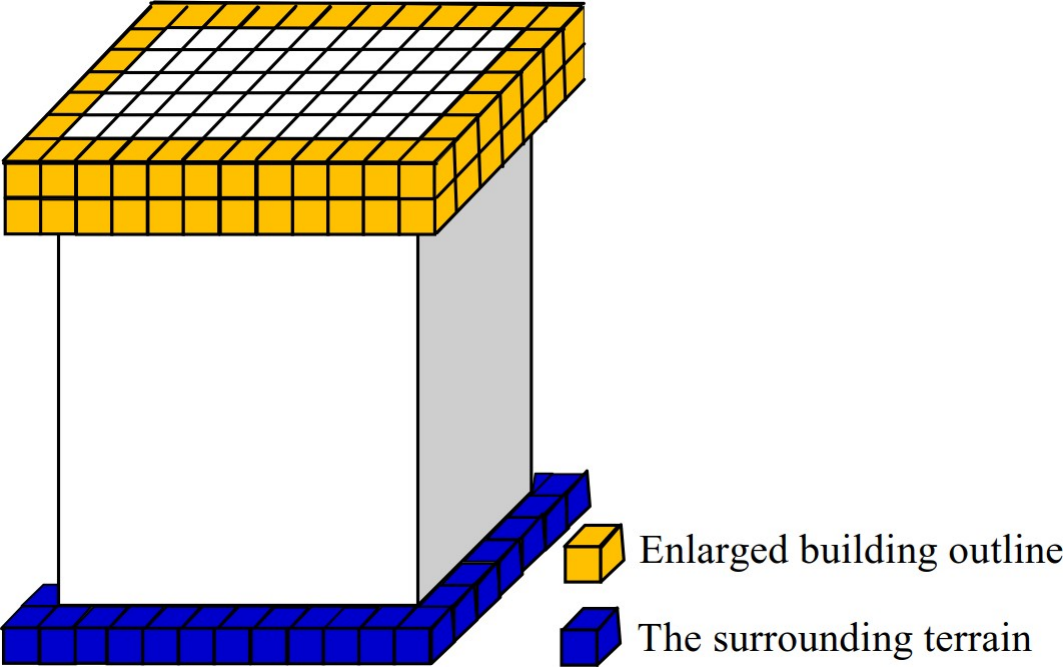
The enlarged building outline and its surrounding terrain of a building.

**Fig 7:**
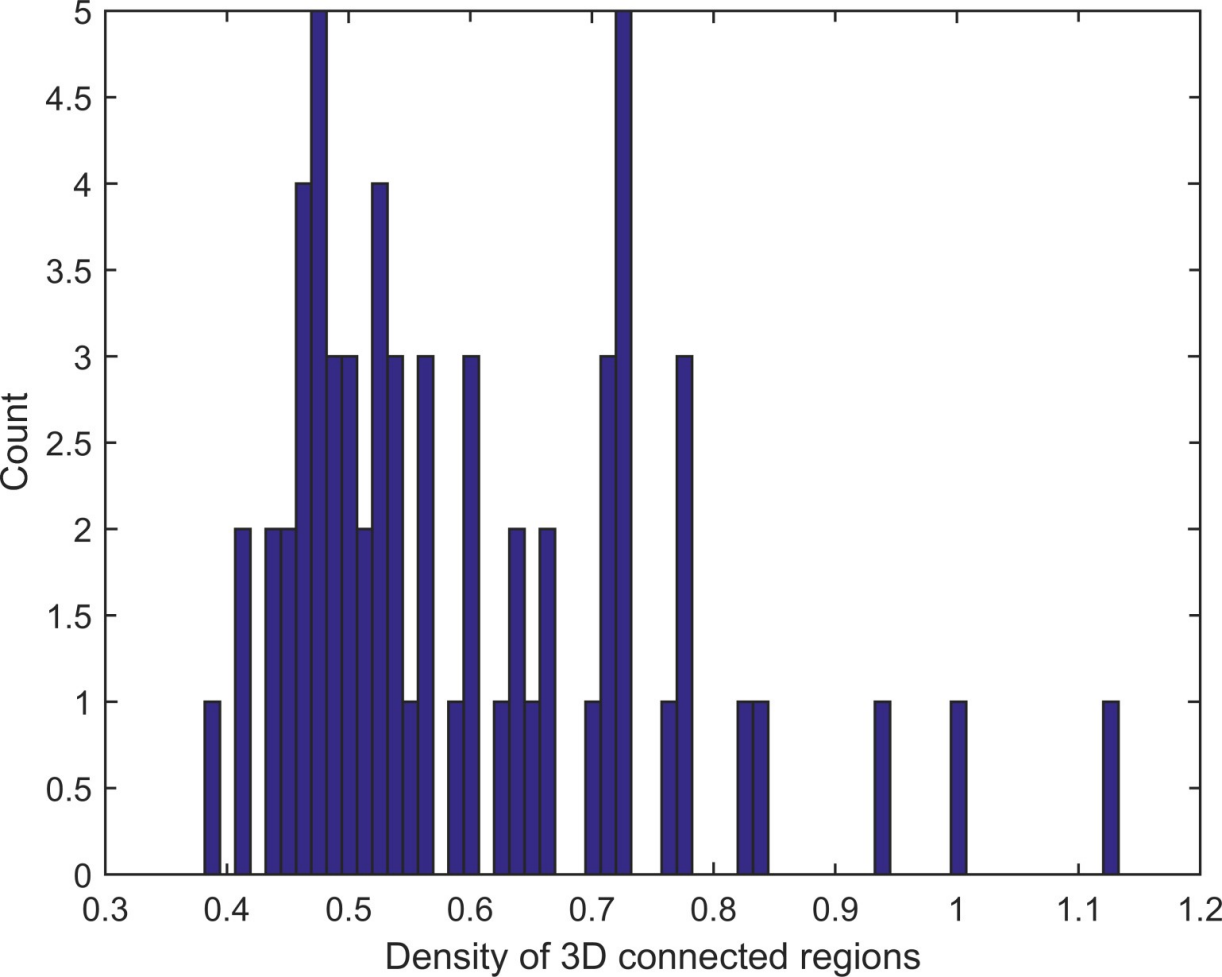
Point density histogram of testing site Area 2.

3D-connected regions corresponding to the building roof can be detected based on their areas, elevation difference and density characteristics. The detailed scheme is as follows. The horizontally projected area of each 3D-connected region is first calculated. If the value is larger than *A*_*min*_ and less than *A*_*max*_ (*A*_*min*_ and *A*_*max*_ denote the areas of the smallest and largest buildings of a given dataset, respectively, which are set according to the real data source and defined by users), the corresponding 3D-connected region is retained as the building roof. Then, the elevation difference between the retained building roof outlines (see the red voxels in Fig 5) and their surrounding terrain (see the blue voxels in Fig 6) is calculated. If the value is larger than the elevation threshold *T*_*e*_ (e.g. 2 m), the corresponding 3D-connected region is retained; otherwise, it is deleted. The elevation of a building roof outline is obtained by calculating the average elevation of the outline voxels. The elevation of the surrounding terrain of a building is obtained based on a 3D morphological dilation operation. 3D morphological dilation with a structuring element [1 1 1; 1 1 1; 1 1 1] is used to enlarge a retained building roof. The outer outline of the enlarged building roof (see the yellow voxels in Fig 6) is a set of voxels and is denoted as *C*_*k*_ *=* {*v*_*t*_ (*r*_*t*_, *c*_*t*_, *l*_*t*_), *t* = 1, …, *q*}, where *k* is the index of the retained buildings, *t* is the index of voxels on the outer outline, and *q* is the total number of voxels within *C*_*k*_. For ∀*v*_*t*_ (*r*_*t*_, *c*_*t*_, *l*_*t*_) ∈*C*_*k*_, the filled voxels that have the same horizontal coordinate as (*r*_*t*_, *c*_*t*_) are searched. The average elevation of the above voxels is used as the elevation of the surrounding terrain of the *k*th retained building roof. Finally, the density of each retained building roof is calculated. If the value is larger than the density threshold *T*_*d*_, the corresponding 3D-connected region is detected as a building roof. *T*_*d*_ can be determined according to point density histogram analysis. A point density histogram is calculated from the retained building roofs and is visualized, as shown in Fig 7. Because laser pulses have a high chance of penetrating holes in a vegetation canopy but cannot penetrate building roofs, the point density of vegetation is lower than that of building roofs, hence the valley (0.68) in the histogram is set as the *T*_*d*_. Algorithm 2 shows the pseudo code of the building roof detection process.

**Algorithm 2**: Pseudo code of the building roof detection process

**Input**: *CR* = 3D-connected regions labeled with *L*_*d*_, 1 ≤ *d* ≤ *k*, *A*_*min*_ and *A*_*max*_ = horizontally projected area of the smallest and largest building of the given dataset, respectively, *T*_*e*_ = 2 (m) (elevation threshold), *T*_*d*_ = density threshold

**Output**: *CR*_*br*_ = 3D-connected regions corresponding to the building roof

**1**    Initialize *CR*_*br*1_ = 0 (3D-connected regions corresponding to the first retained building roofs)

**2**    **for** *i′* = 1 **to** *k* do

**3**            Calculate the horizontally projected area *A*_1_ of *i′* th *CR*

**4**            **if** *A*_1_∈ [*A*_*min*_, *A*_*max*_] **then** *CR*_*br*1_ = *CR*_*br*1_+ *i*′ th *CR*

**5**    **end**

**6**    Set *n*_*br1*_ = #*CR*_*br1*_

**7**    Initialize *CR*_*br*2_ = 0 (3D-connected regions corresponding to the second retained building roofs)

**8**    **for** *j′* = 1 **to** *n*_*br*1_ do

**9**            Calculate the elevation difference *e*_*d*_ between the *j′* th *CR*_*br*1_ outline and its surrounding terrain

**10**            **if** *e*_*d*_ > *T*_*e*_ **then** *CR*_*br*2_ = *CR*_*br*2_+ *j′* th *CR*_*br*1_

**11**    **end**

**12**    Set *n*_*br*2_ = #*CR*_*br2*_

**13**    Initialize *CR*_*br*_ = 0

**14**    **for** *k′* = 1 **to** *n*_*br*2_ do

**15**            Calculate the density *D*_1_ of *k′* th *CR*_*br*2_

**16**            **if** *D*_1_ > *T*_*d*_ **then** CR_br_ = CR_br_ + *k*′ th CR_br2_

**17**    **end**

**Fig 8:**
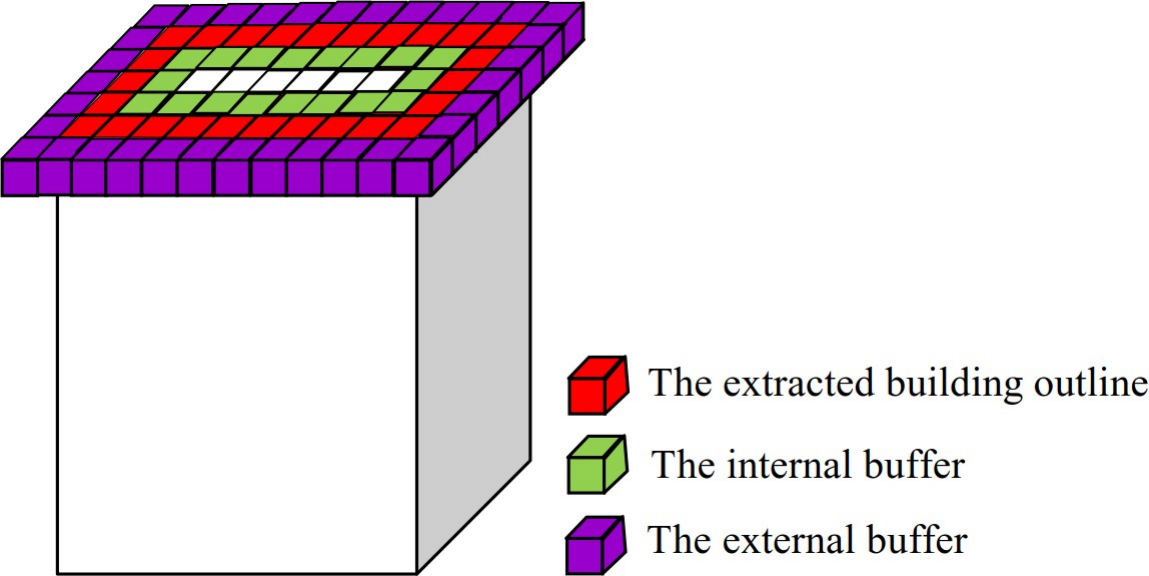
Buffer setting.

3D-connected regions corresponding to the building façade are detected according to the following characteristics: the building façade is usually vertical to its corresponding building outline and is located within a certain range of its corresponding building outline. Accordingly, the building outline of each 3D-connected region corresponding to the building roof is first extracted (see the red voxels in Fig 8). Then, 3D-connected regions that fall in buffers (see the green and purple voxels in Fig 8) centered at the projection of the building outline with two voxels on both sides of the building outline with similar grayscales to that of the corresponding building outline (that is, their grayscales are within the statistical range of the building) are detected as the building facade. Algorithm 3 shows the pseudo code of the building facade detection process.

**Algorithm 3**: Pseudo code of the building facade label process

**Input**: *CR*_*br*_ = 3D-connected regions corresponding to the building roof, *CR*_*nbr*_ = 3D-connected regions that do not correspond to the building roof, *R*_*l*_ and *R*_*r*_ *=* the grayscale range of the building

**Output**: *CR*_*bf*_ = 3D-connected regions corresponding to the building facade

**1**    Set *n*_*br*_ = #*CR*_*br*_

**2**    Set *b*_*s*_ = 0 (the buffers of the extracted building roof outline)

**3**    **for** *i*_1_ = 1 **to** *n*_*br*_ do

**4**            Extracte the building roof outline *b*_*ro*_ of *i*_1_th *CR*_*br*_ and determine the buffer *b*_*e*_ of *b*_*ro*_

**5**            *b*_*s*_ = *b*_*s*_ + *b*_*e*_

**6**    **end**

**7**    Set *n*_*nbr*_ = # *CR*_*nbr*_

**8**    Set *CR*_*bf*_ = 0

**9**    **for** *j*_1_ = 1 **to** *n*_*nbr*_ do

**10**            **if** the *j*_1_th *CR*_*nbr*_ falls in *b*_*s*_ and their grayscales ∈ [*R*_*l*_, *R*_*r*_] **then** *CR*_*bf*_ = *CR*_*bf*_ *+j*_1_th *CR*_*nbr*_

**11**    **end**

### Evaluation

The detected results of the proposed VSBD algorithm are represented as building voxels, and the referenced data are discrete LIDAR building points. To compare the results from the proposed VSBD algorithm with those of the reference data, the discrete LIDAR points included in the detected building voxels are obtained, then the extracted buildings and the reference buildings are compared point-by-point. Based on the comparison result between the two datasets, the following accuracy indexes [46] were employed to quantitatively assess the proposed VSBD algorithm:

$$\begin{array}{l} Type\;I\;error=\frac{FN}{TP+FN}\;Type\;II\;error=\frac{FP}{FP+TN}\;Total\;error=\frac{FN+FP}{TP+FN+FP+TN}\\ Kappa=\frac{P_{0}-P_{e}}{1-P_{e}}\;P_{0}=\frac{TP+TN}{TP+FN+FP+TN}\;P_{e}=\frac{\left(TP+FN)\times (TP+FP)+(FP+TN)\times (FN+TN\right)}{{\left(TP+FN+FP+TN\right)}^{2}}\\ Completeness=\frac{TP}{TP+FN}\;Correctness=\frac{TP}{TP+FP}\;Quality=\frac{TP}{TP+FN+FP}. \end{array}$$ (3)

where Type I error is the percentage of building points rejected as nonbuilding points, Type II error is the percentage of nonbuilding points accepted as building points, Total error is the percentage of incorrectly classified points, Completeness is the percentage of reference data being detected, Correctness is the percentage of correct detection, Quality is the overall success rate, the Kappa coefficient is a statistical measure of the interratio agreement, which is believed to be a more robust measurement than a simple percentage, *TP* (True Positive) is the number of building points classified by both datasets, *TN* (True Negative) is the number of nonbuilding points classified by both datasets, *FP* (False Positive) is the number of building points classified only by the proposed VSBD algorithm, *FN* (False Negative) is the number of building points classified only by the reference dataset.

## Results

### Experimental results and discussions

**Fig 9:**
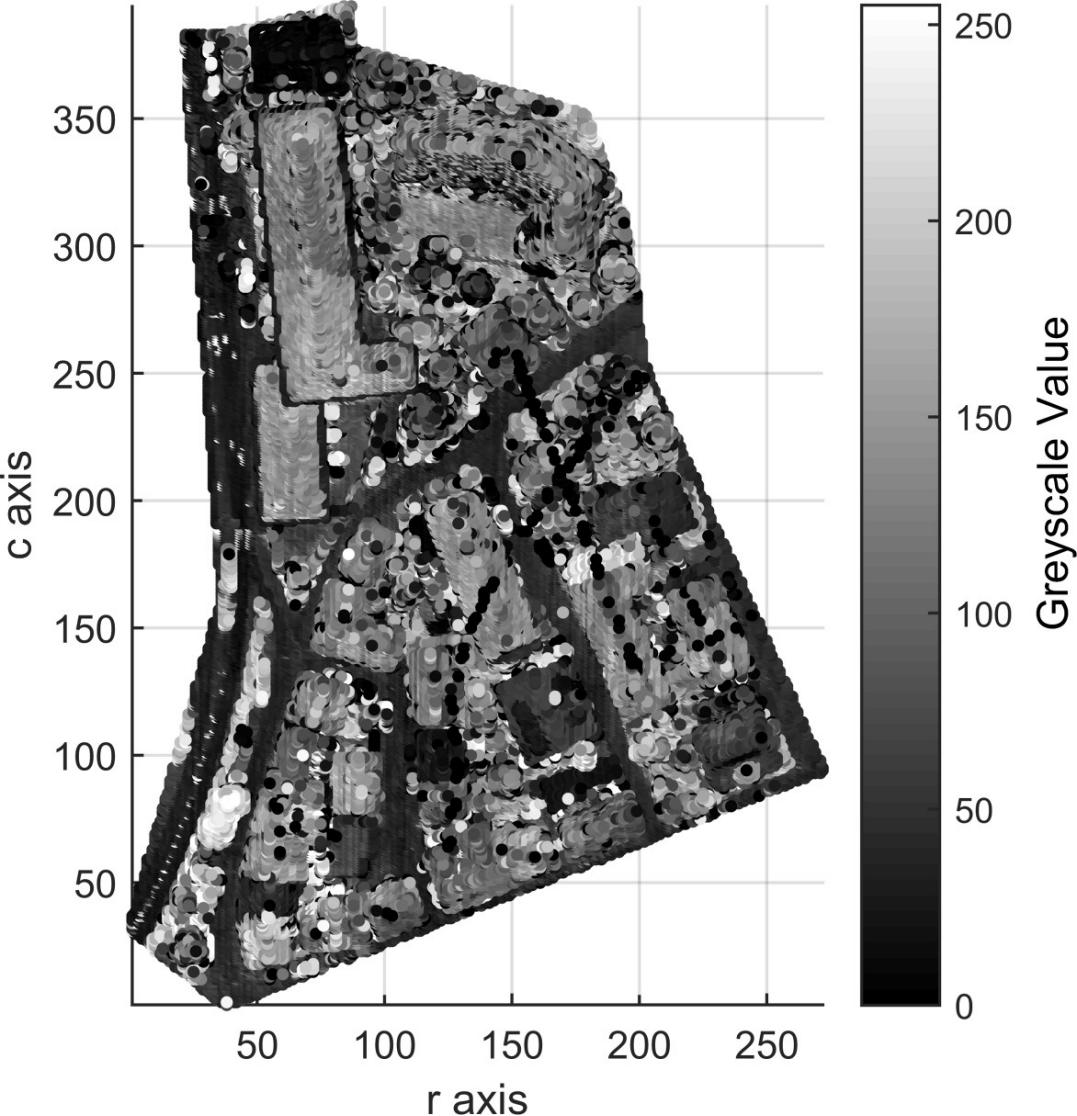
Top views of the voxelized datasets of Area 1.

**Fig 10:**
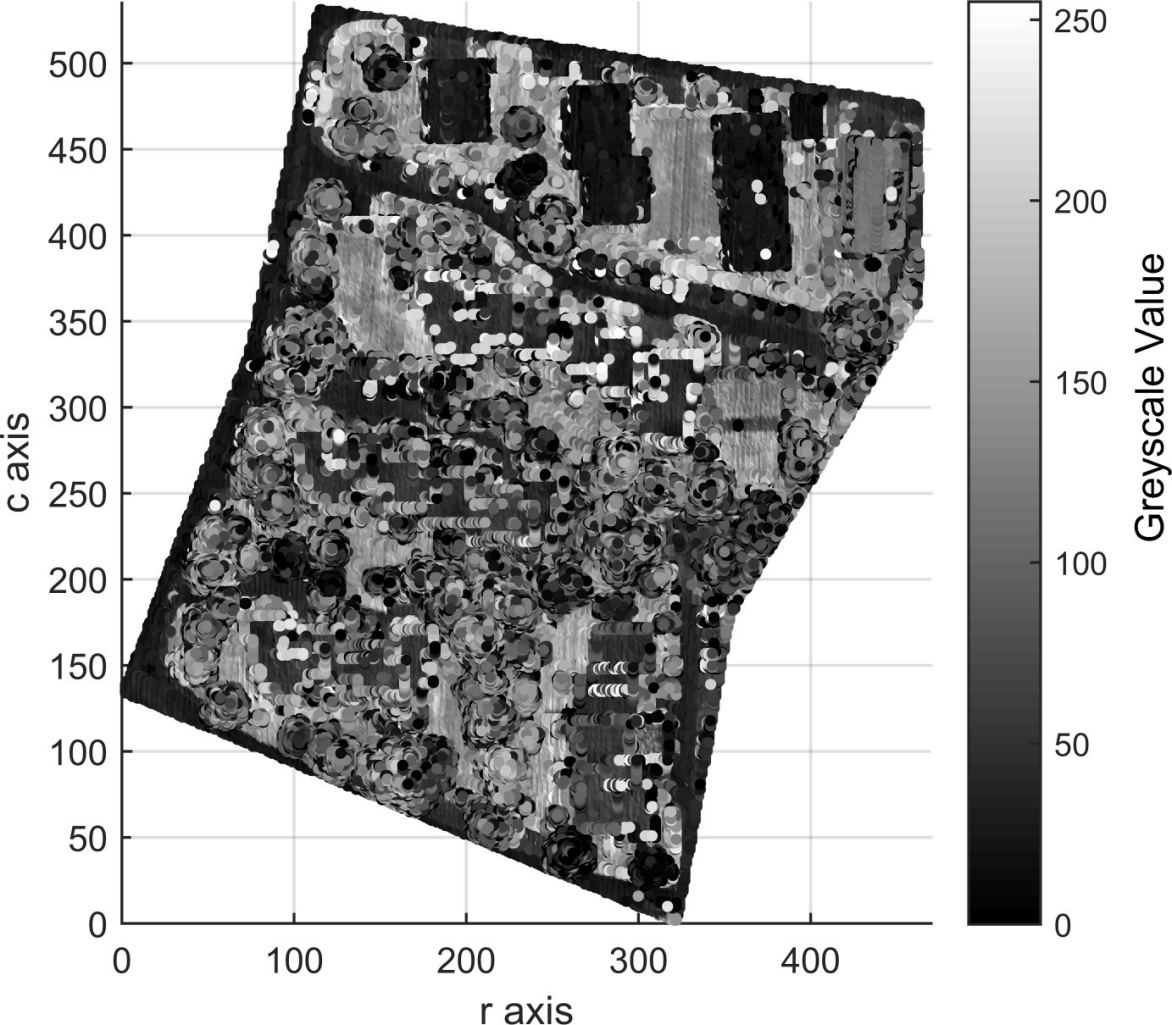
Top views of the voxelized datasets of Area 2.

**Fig 11:**
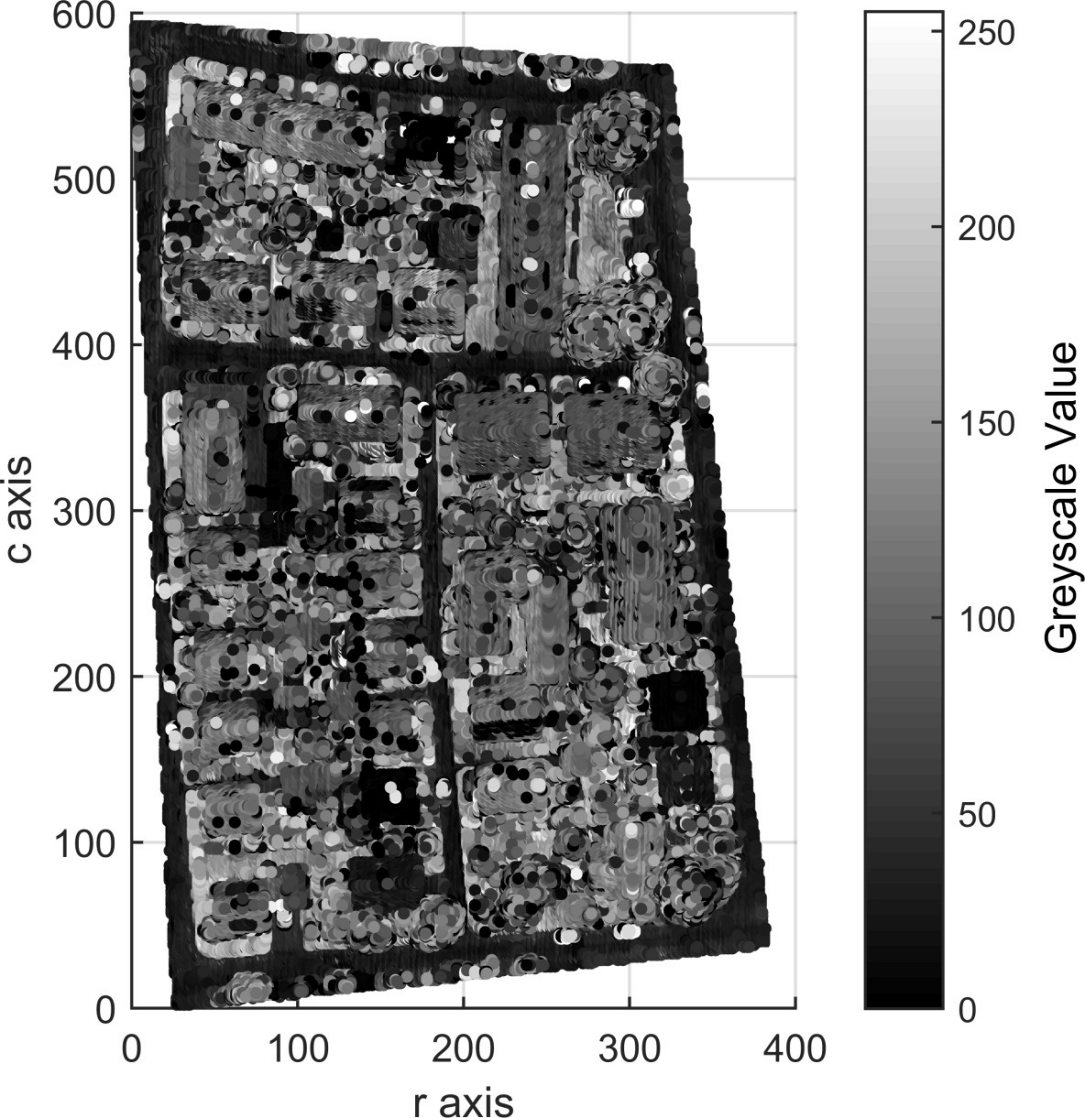
Top views of the voxelized datasets of Area 3.

Areas 1, 2 and 3 consists of 104,188, 243,127 and 237,875 points, respectively, which contain 0, 0 and 2 outliers, respectively. After outlier removal, the data are remapped into 3D arrays measuring 272 × 395 × 60 for Area 1, 463 × 531 × 98 for Area 2 and 382 × 593 × 63 for Area 3. Figs 9, 10 and 11 show top views of the voxelized datasets with a voxel resolution of 0.5 m^3^ for Area 1 and 0.4 m^3^ for Areas 2 and 3; 61121, 154566 and 150098 filled voxels were obtained for Areas 1, 2 and 3, respectively.

The grayscales of buildings and other objects in Figs 9, 10 and 11 can be used as the prior knowledge to determine the statistical grayscale ranges of objects in the subsequent segmentation process.

The segmentation process is implemented to group filled voxels into multiple 3D-connected regions. As noted above, the segmentation and building detection results are related to the statistical grayscale ranges of the objects and the adjacency size.

The grayscale ranges of the objects were determined using the scheme described in the“Segmentation of voxelized dataset” section and are listed in Table 1 for the three testing sites.

**Table 1:** The statistical grayscale ranges of objects for all testing sites.

Testing sites	Grayscale range of each Gauss distribution			
	First Gauss distribution	Second Gauss distribution	Third Gauss distribution	Fourth Gauss distribution
**Area 1**	(0, 11]	(11, 39)	[39, 255]	-
**Area 2**	(0, 4)	[4, 90]	(90, 255]	-
**Area 3**	(0, 10]	(10, 61)	[61, 103]	(103,255]

To determine the effects of adjacent sizes on the building detection results and the optimal adjacent size, 6-, 18-, 26-, 56- and 80-adjacency were used for each testing site under identical conditions and the corresponding accuracy indexes are listed in Table 2.

**Table 2:** Accuracy indexes of different adjacency sizes for all testing sites.

Testing site	Adjacency	Kappa (%)	Type I Error (%)	Type II Error (%)	Total Error (%)
**Area 1**	6	17.2	85.2	0.6	31.9
**Area 1**	18	52.5	51.9	1.0	19.9
**Area 1**	26	62.9	39.2	2.3	16.2
**Area 1**	56	88.0	9.4	3.3	5.6
**Area 1**	80	80.5	9.7	9.3	9.4
**Area 2**	6	52.7	56.7	0.3	14.5
**Area 2**	18	83.8	21.1	0.5	5.7
**Area 2**	26	85.0	20.2	0.3	5.3
**Area 2**	56	95.0	6.2	0.4	1.9
**Area 2**	80	94.4	5.2	1.1	2.1
**Area 3**	6	16.3	83.7	1.5	37.0
**Area 3**	18	49.2	51.5	1.6	24.0
**Area 3**	26	57.6	42.1	2.4	20.2
**Area 3**	56	83.1	14.5	3.4	8.2
**Area 3**	80	80.2	12.3	7.7	9.7

As listed in Table 2, the average Kappa coefficients for the 6-, 18-, 26-, 56-, and 80-adjacency are 28.7%, 61.8%, 68.5%, 88.7% and 85.1%, respectively, which means that using the 56-adjacency generates the maximum Kappa coefficient. Consequently, the 56-adjacency can be considered the optimal adjacency size considering the Kappa coefficient. The average Total errors for the 6-, 18-, 26-, 56- and 80-adjacency are 27.8%, 16.5%, 13.9%, 5.2% and 7.1%, respectively, which means that using the 56-adjacency generates the minimum Total error and the 56-adjacency is also the optimal adjacency size when considering the Total error.

Moreover, accuracies do not always increase with increasing adjacency sizes. The idea behind the proposed VSBD algorithm is that object information (e.g., building) can be passed through a GVS based on the connectivity and grayscale similarity defined in the 3D array. Taking 6-adjacency as an example, propagation of object information can only move from the center voxel up, down, or in the four cardinal directions based on the grayscale attribute associated with each voxel. The 6-adjacent LABEL can work well for flat-roofed buildings (e.g., Area 2) where rooftop voxels can be merged into one 3D-connected region and be correctly detected whereas it works relatively poorly for peak-roofed buildings (e.g., Areas 1 and 3) where rooftop voxels may be grouped into multiple 3D-connected regions and be taken as nonbuildings using area or elevation jump characteristics. This explains why 6-adjacency has much better performance in Area 2 than in Areas 1 and 3. With increasing adjacency size, propagation of object information through the 3D array with 18-, 26- or 56-adjacent connectivity increases the directions of spread and more voxels are likely to be considered and thus improves the detection accuracy. This may be why the 18-, 26- and 56-adjacent LABELs have much better performance than the 6-adjacent LABEL. However, if the adjacency size is too large, some nonbuilding voxels may be taken as building voxels and increase the Type II error. This may be why the accuracy declines when the 80-adjacent LABEL is used.

**Fig 12:**
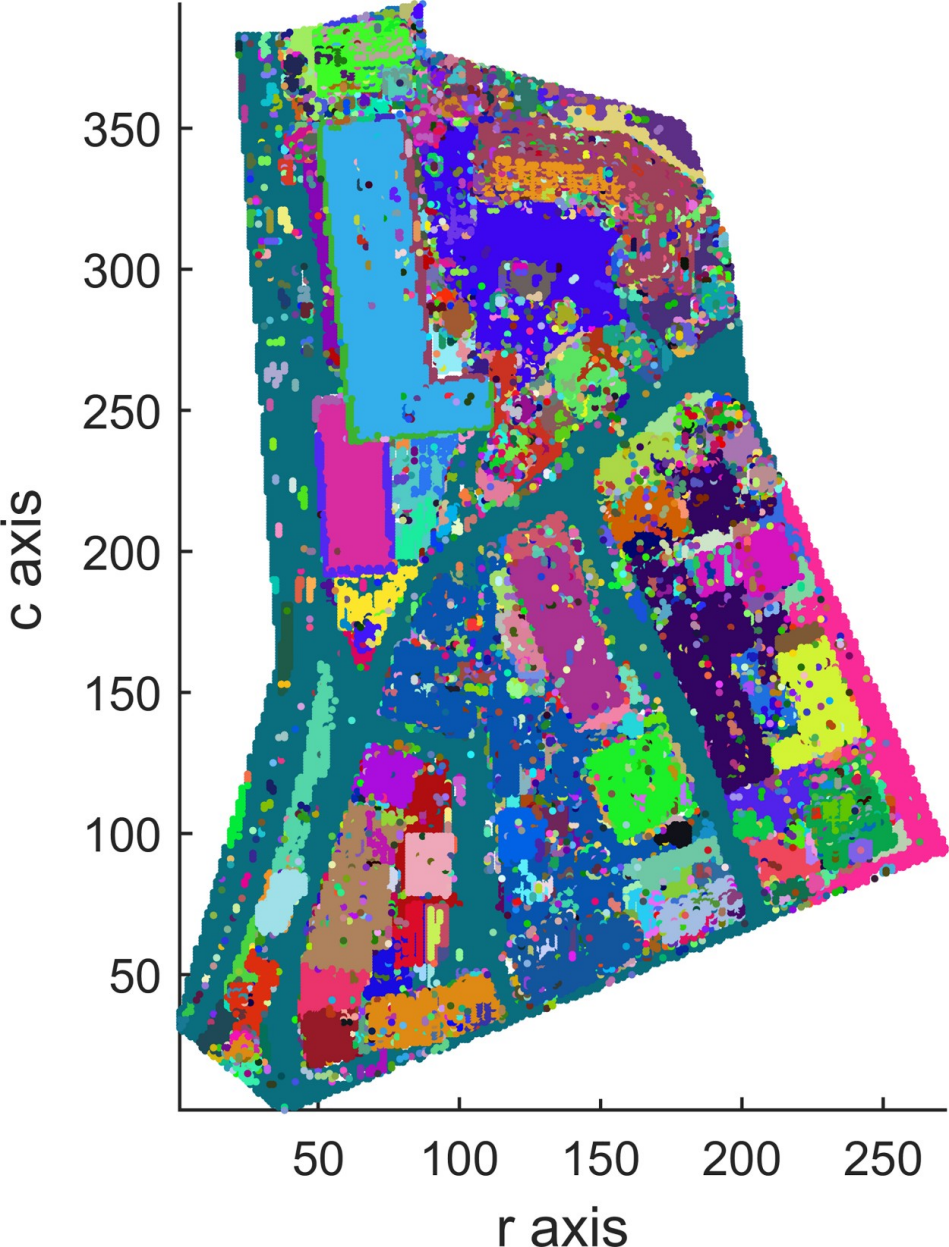
Top view of the segmentation result for Area 1 (3D-connected regions are denoted using different colors).

**Fig 13:**
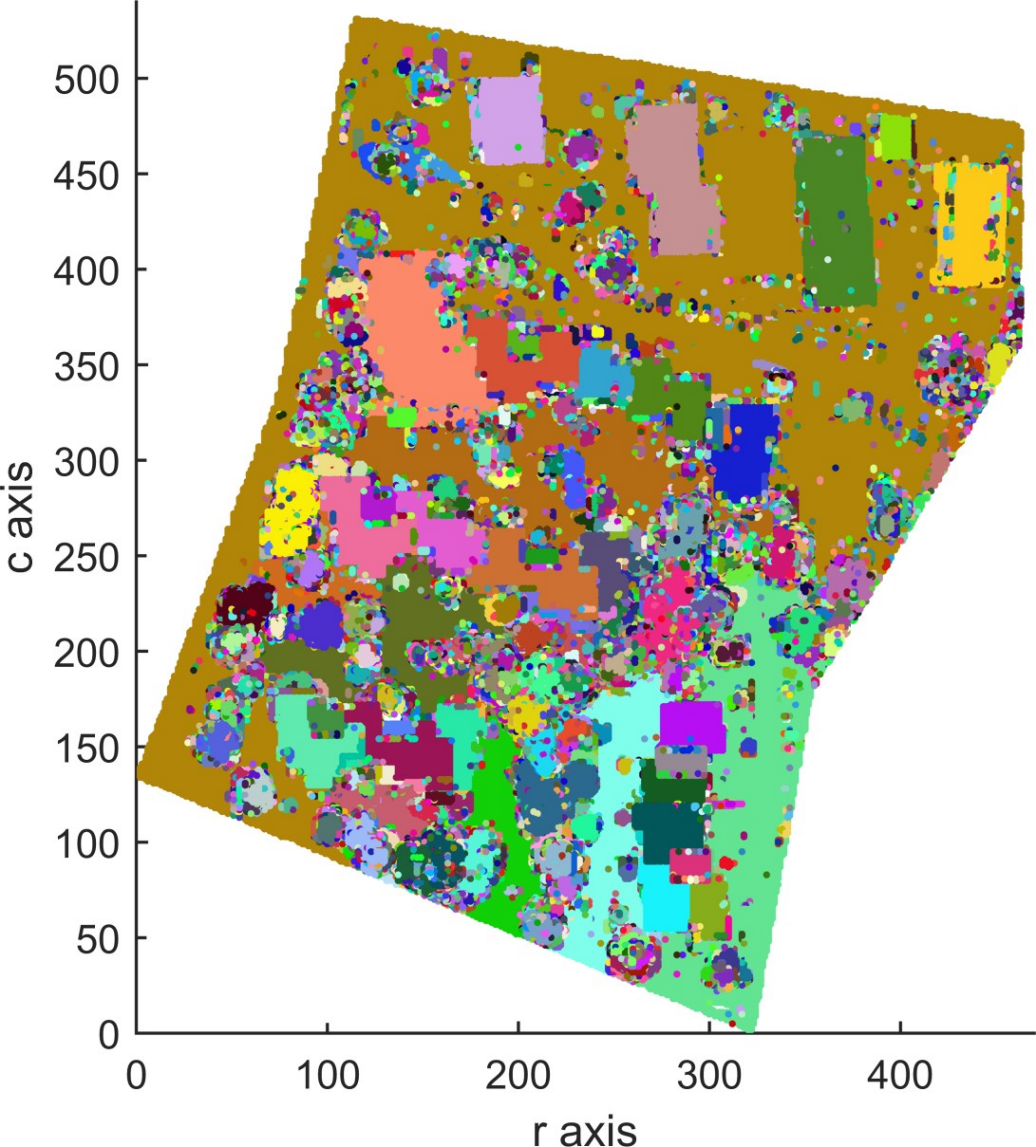
Top view of the segmentation result for Area 2 (3D-connected regions are denoted using different colors).

**Fig 14:**
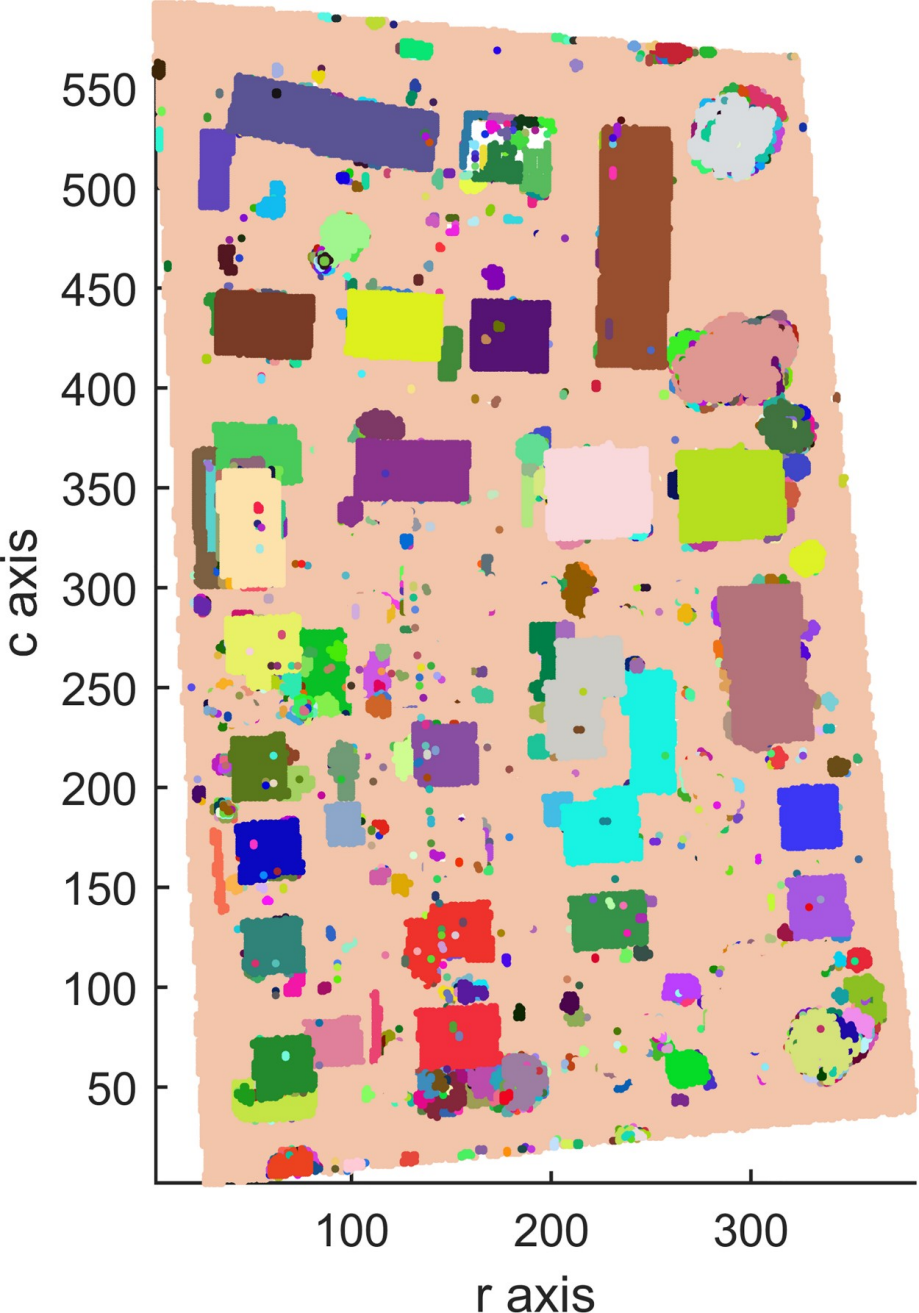
Top view of the segmentation result for Area 3 (3D-connected regions are denoted using different colors).

Top views of the segmentation results of Areas 1, 2 and 3 with the optimal 56-adjacency are shown in Figs 12, 13 and 14, respectively.

As shown in Figs 12, 13 and 14, all building objects are divided into separate 3D-connected regions. The problem of oversegmentation is apparent (see the white area in Fig 14).

**Fig 15:**
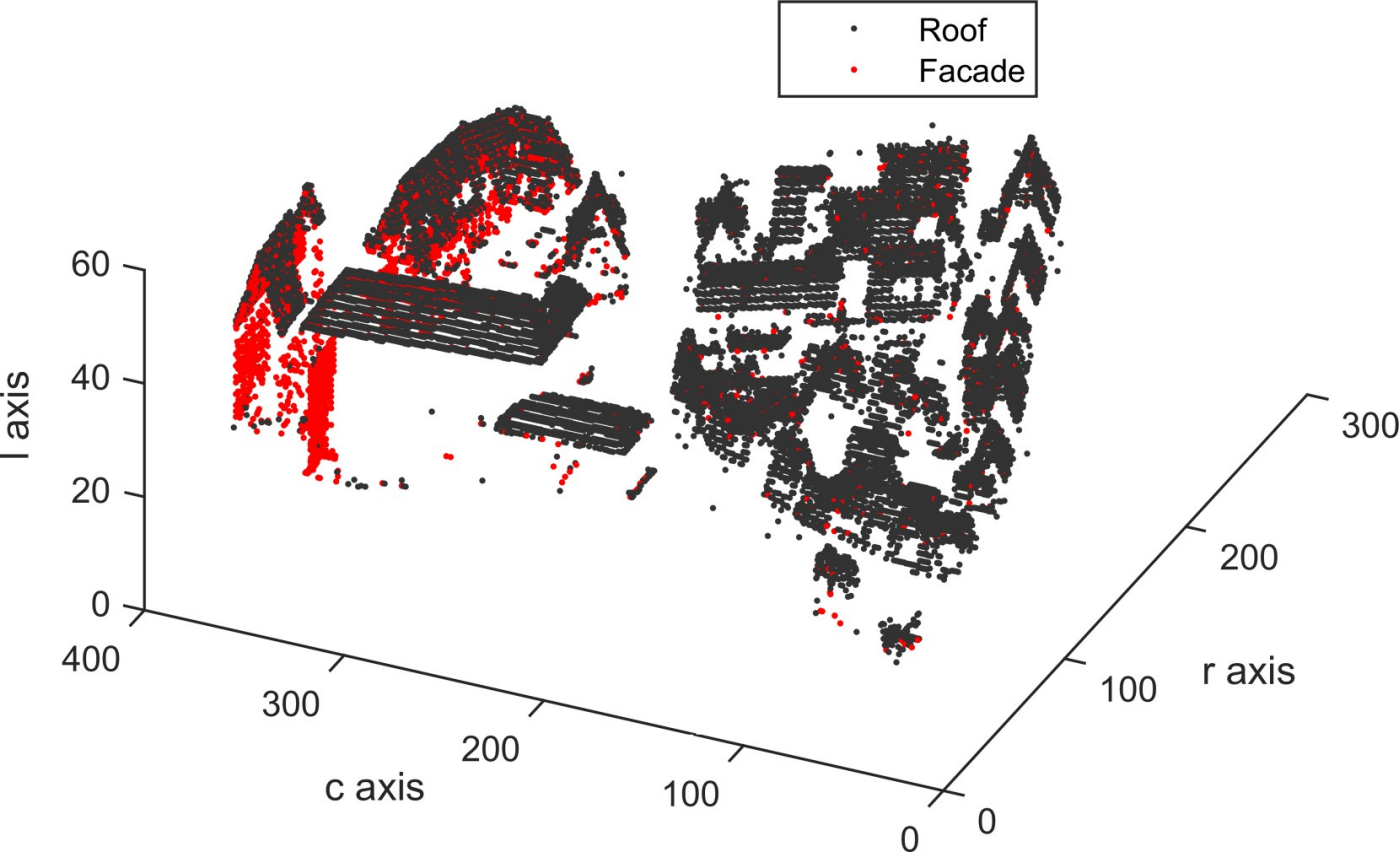
Detected building voxels for Area 1.

**Fig 16:**
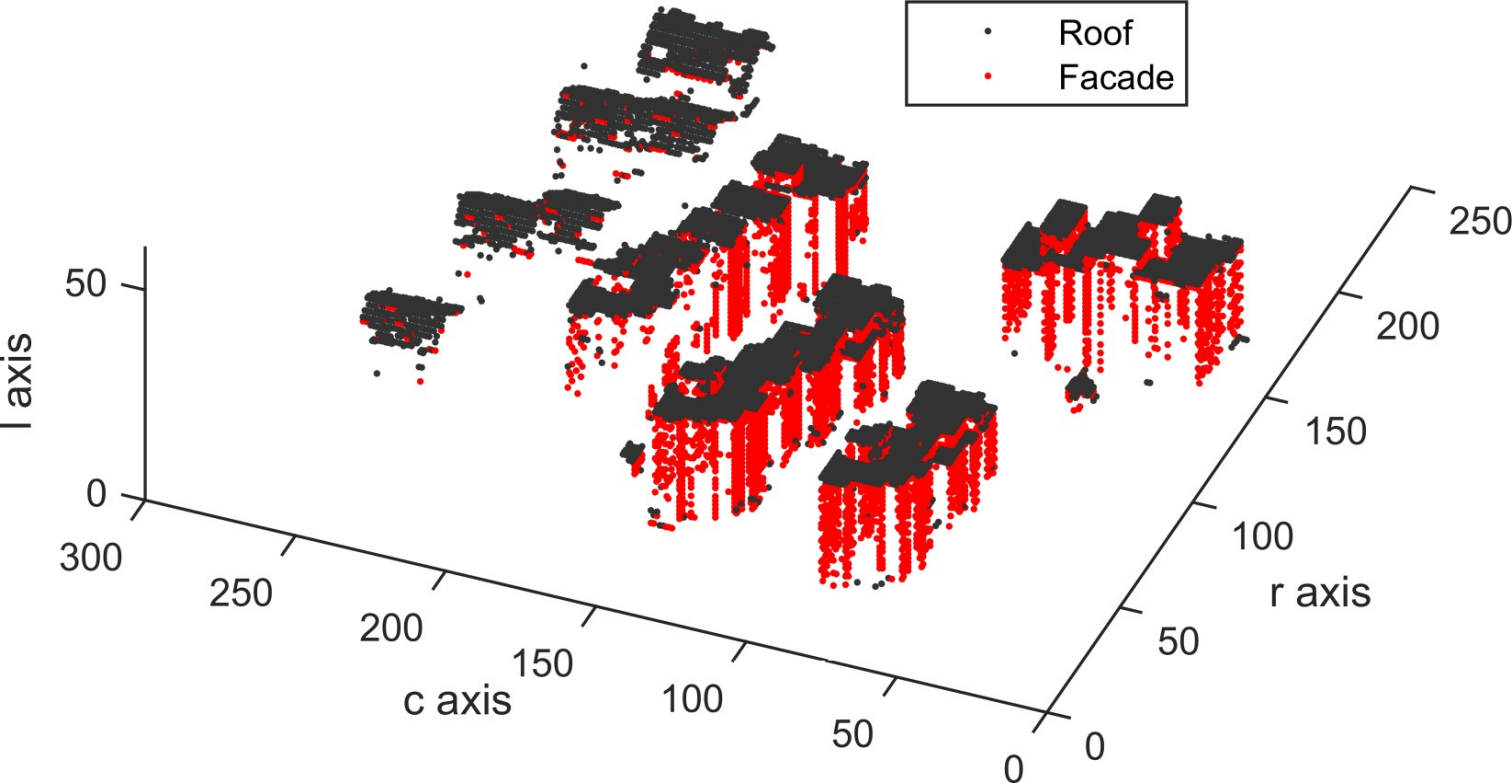
Detected building voxels for Area 2.

**Fig 17:**
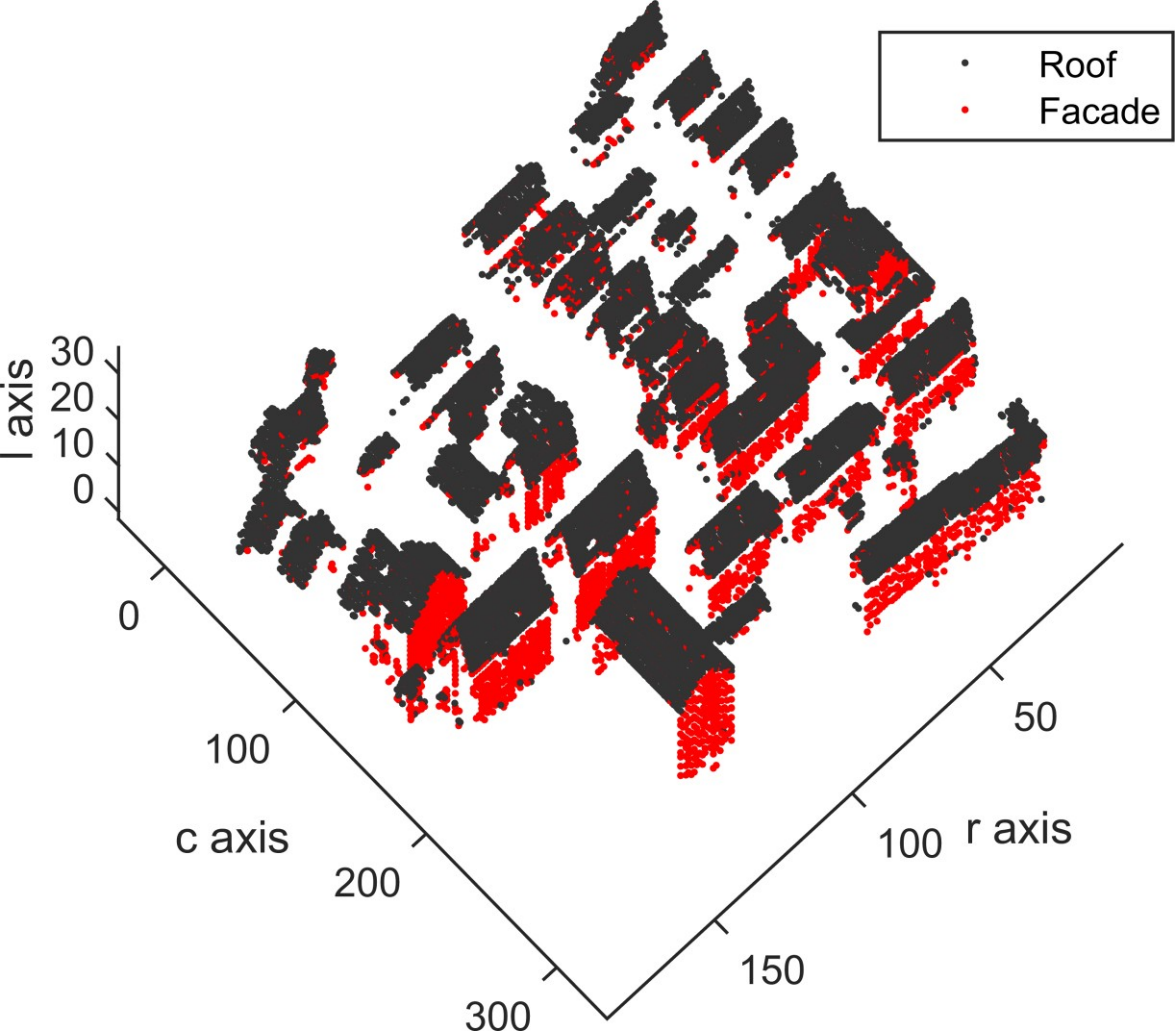
Detected building voxels for Area 3.

Building roof and facade detection are implemented, and the detected results of Areas 1, 2 and 3 are shown in Figs 15, 16 and 17, which contain 22935, 25933 and 37589 building voxels, respectively. The above detected building results can directly serve as a 3D building model in a the form of a voxel model.

The building detection results of the proposed VSBD algorithm are determined by input parameters such as thresholds (*A*_*min*_, *A*_*max*_, *T*_*e*_ and *T*_*d*_), the statistical grayscale ranges of objects and the adjacency size. The statistical grayscale ranges of objects, *A*_*min*_, *A*_*max*_ and *T*_*d*_ are set according to the real data source. The data source thresholds are easily determined using the solutions given in this paper, allowing for application of the proposed VSBD algorithm in other areas for building detection. *T*_*e*_ is set empirically as 2 m because buildings are at least 2m above the surrounding ground. The adjacency size can use 56-adjacency directly because it is the optimal adjacency size in areas with diverse building types. Therefore, the proposed VSBD algorithm can be applied in other urban scenes as a suitable method for the detection of 3D buildings.

### Quantitative assessment

A quantitative accuracy assessment was performed to evaluate the performance of the proposed VSBD algorithm using the optimal 56-adjacency (see Table 3).

**Table 3:** Evaluation results of detected buildings in per-area mode for all testing sites.

Testing site	Completeness (%)	Correctness (%)	Quality(%)
**Area 1**	90.6	94.5	86.0
**Area 2**	93.8	98.7	92.7
**Area 3**	85.5	94.9	85.7
**Average**	90.0	96.0	88.1

**Fig 18:**
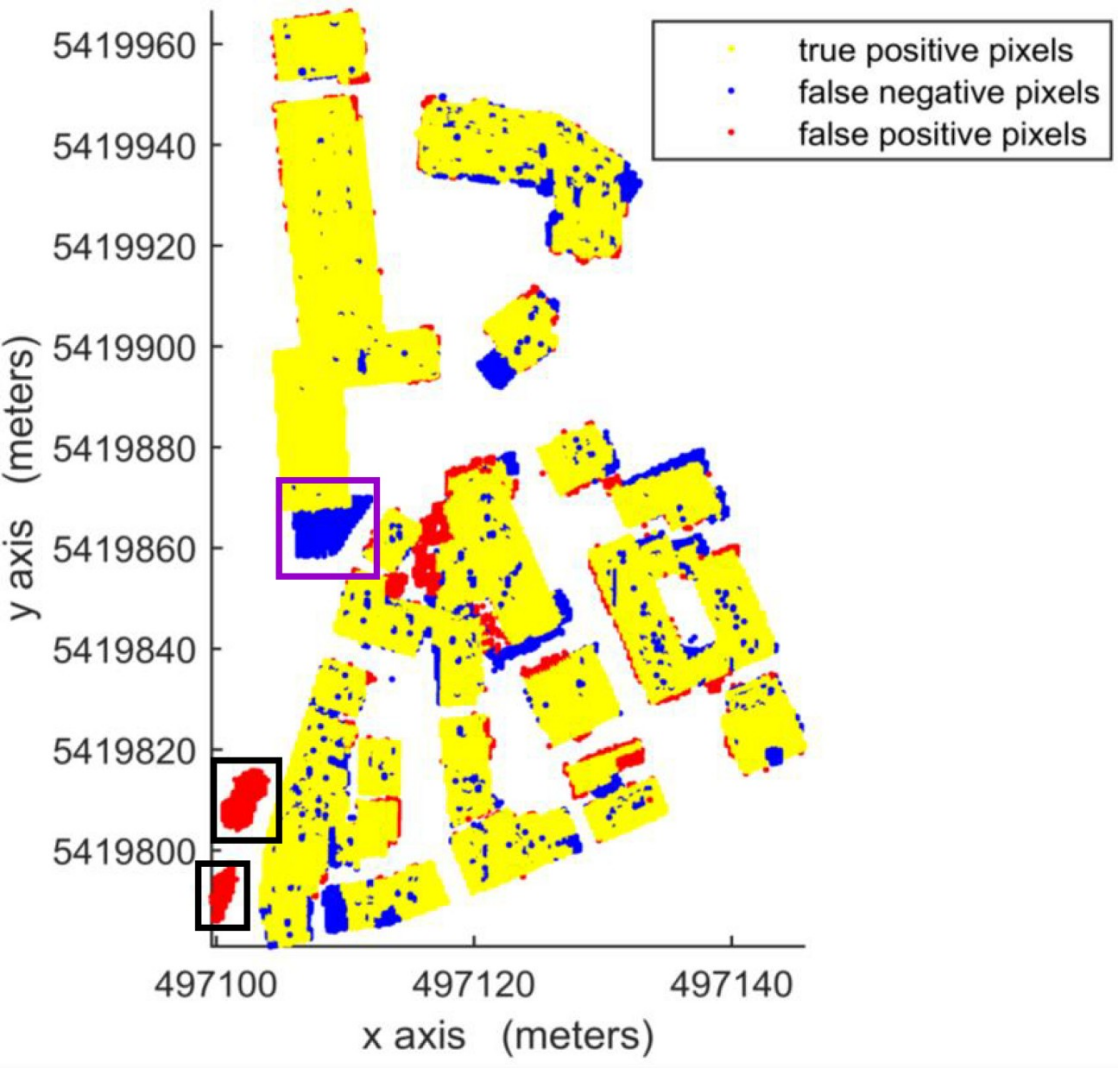
Top view of the building detection results and errors of the proposed VSBD algorithm for Aera 1.

**Fig 19:**
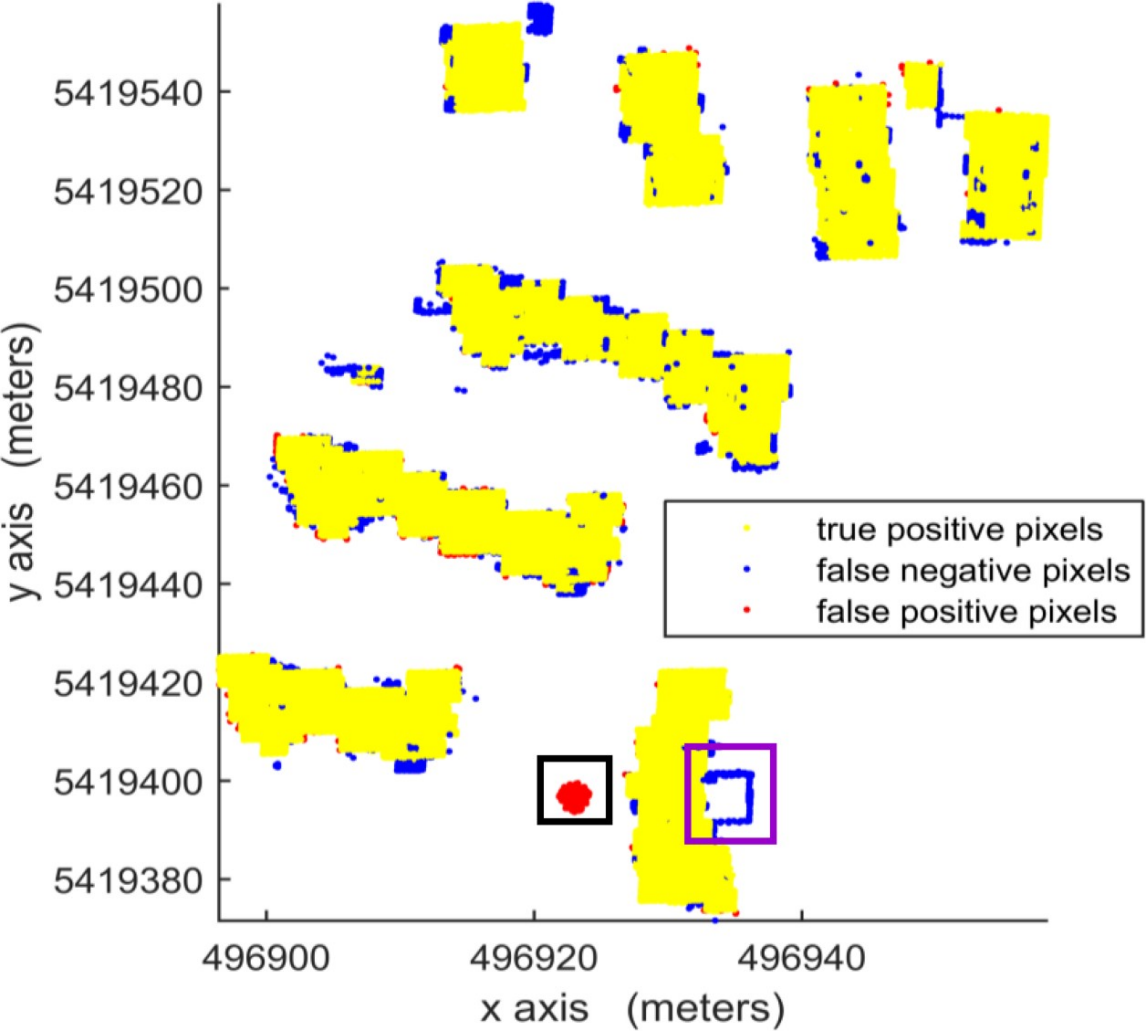
Top view of the building detection results and errors of the proposed VSBD algorithm for Aera 2.

**Fig 20:**
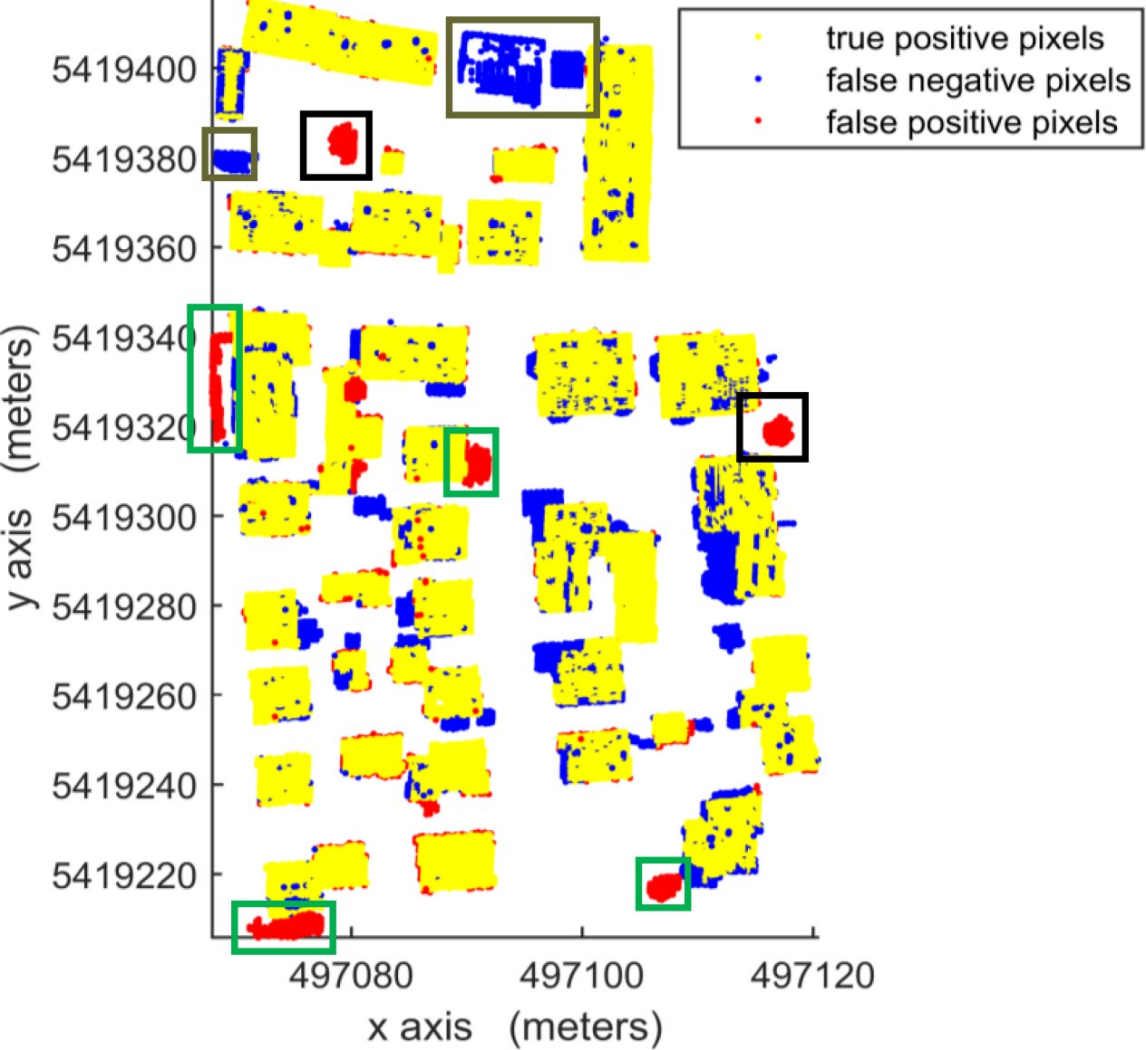
Top view of the building detection results and errors of the proposed VSBD algorithm for Aera 3.

According to Table 3, in per-area mode, an average completeness, correctness and quality of 90.0%, 96.0% and 88.1%, respectively, were obtained for building detection. The proposed VSBD algorithm performs better in Area 2 than in Areas 1 and 3. To explore the origin of the incompleteness and incorrectness, top views of the detected buildings and the distribution of errors for all testing sites are shown in Figs 18, 19 and 20.

Figs 18, 19 and 20 show that almost all buildings were detected successfully. Thus, the proposed VSBD algorithm works well for detecting buildings. Figs 18, 19 and 20 also show that the major factors of incorrectness are as follows. First, some nonbuildings nearby buildings and similar to buildings in grayscale may be taken as buildings, see the green rectangles in Fig 20. Second, some nonbuildings may be taken as buildings if only the area, elevation difference and density characteristics of building are utilized to identify the 3D-connected regions corresponding to the building roof (see the black rectangles in Figs 18, 19 and 20). The major factors of incompleteness are as follows. First, buildings with a very low point density are divided into multiple 3D-connected regions and are missed according to the area or elevation difference criterion. That is, the area of each 3D-connected region is out of the range [*A*_*min*_, *A*_*max*_] or the distance between each 3D-connected region and its surrounding terrain is not less than 2m (see the brown rectangles in Fig 20). Second, some wing-rooms or low buildings, which have similar grayscales to their surrounding ground and form a 3D-connected region with area larger than *A*_*max*_, are also missed (see the purple rectangles in Figs 18, 19 and 20).

Moreover, the effects of GVS constructed with different vertical resolutions Δ*z* on the building detection accuracy of the proposed VSBD algorithm were studied. If Δ*z* is set using Eq (1), the voxel resolution of the constructed GVS is 0.5 m × 0.5 m × 0.1 m for Area 1 and 0.4 m × 0.4 m × 0.1 m for Areas 2 and 3, and the scene volume of testing sites Areas 1, 2 and 3 are divided in 272 × 395 × 293, 463 × 531 × 386 and 382 × 593 × 243 arrays, respectively. The corresponding building detection accuracy using the 56-adjacency size was calculated and the results are presented in Table 4.

**Table 4:** Perarea accuracies f of the proposed VSBD algorithm for detected buildings with the vertical voxel resolution scheme of Eq (1).

Testing Site	Completeness (%)	Correctness (%)	Quality (%)
**Area 1**	82.6	90.6	76.1
**Area 2**	88.2	98.1	86.7
**Area 3**	45.3	94.6	44.2
**Average**	72.0	94.4	69.0

**Fig 21:**
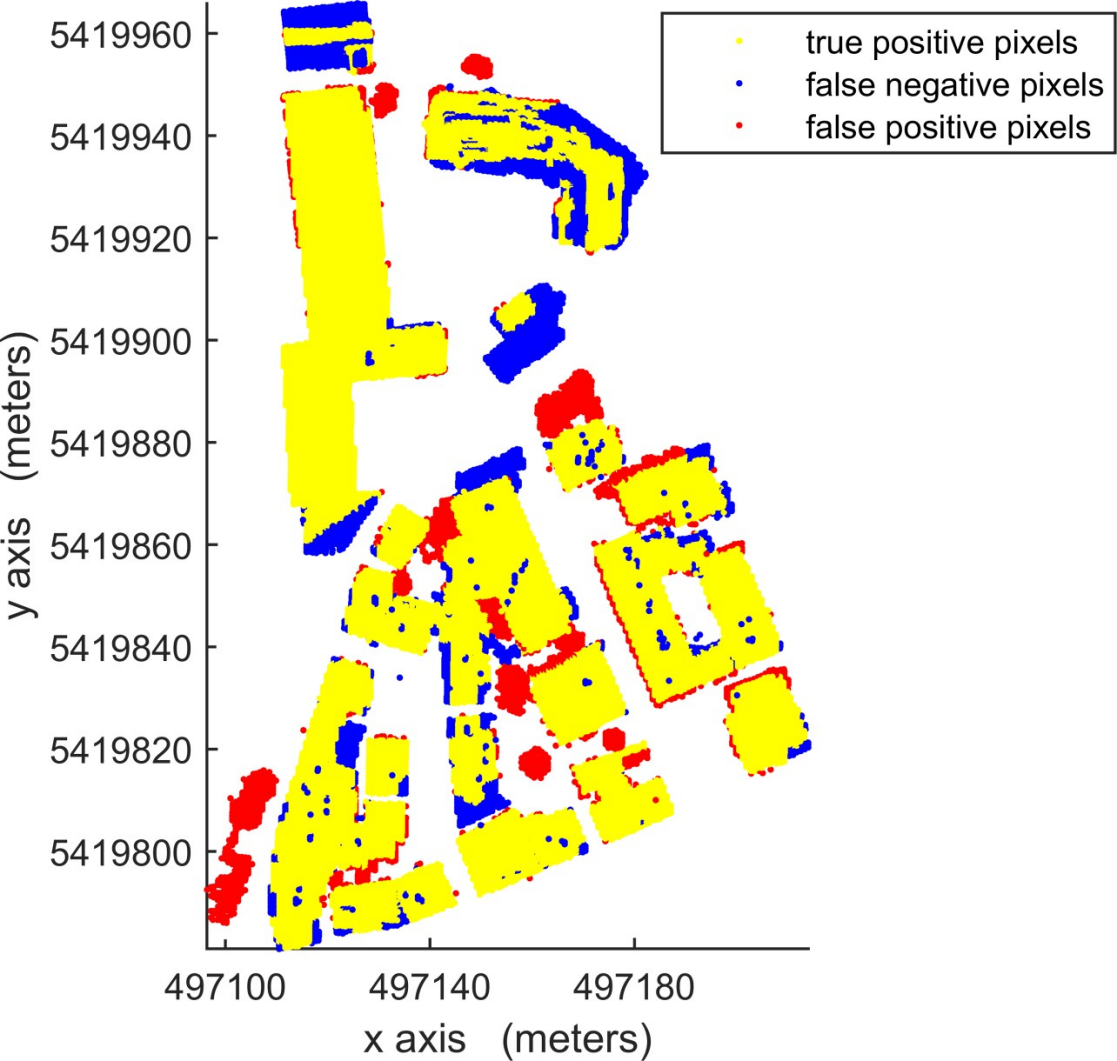
Top view of the building detection results and errors of the proposed VSBD algorithm for Area 1 with the vertical voxel resolution scheme of Eq (1).

**Fig 22:**
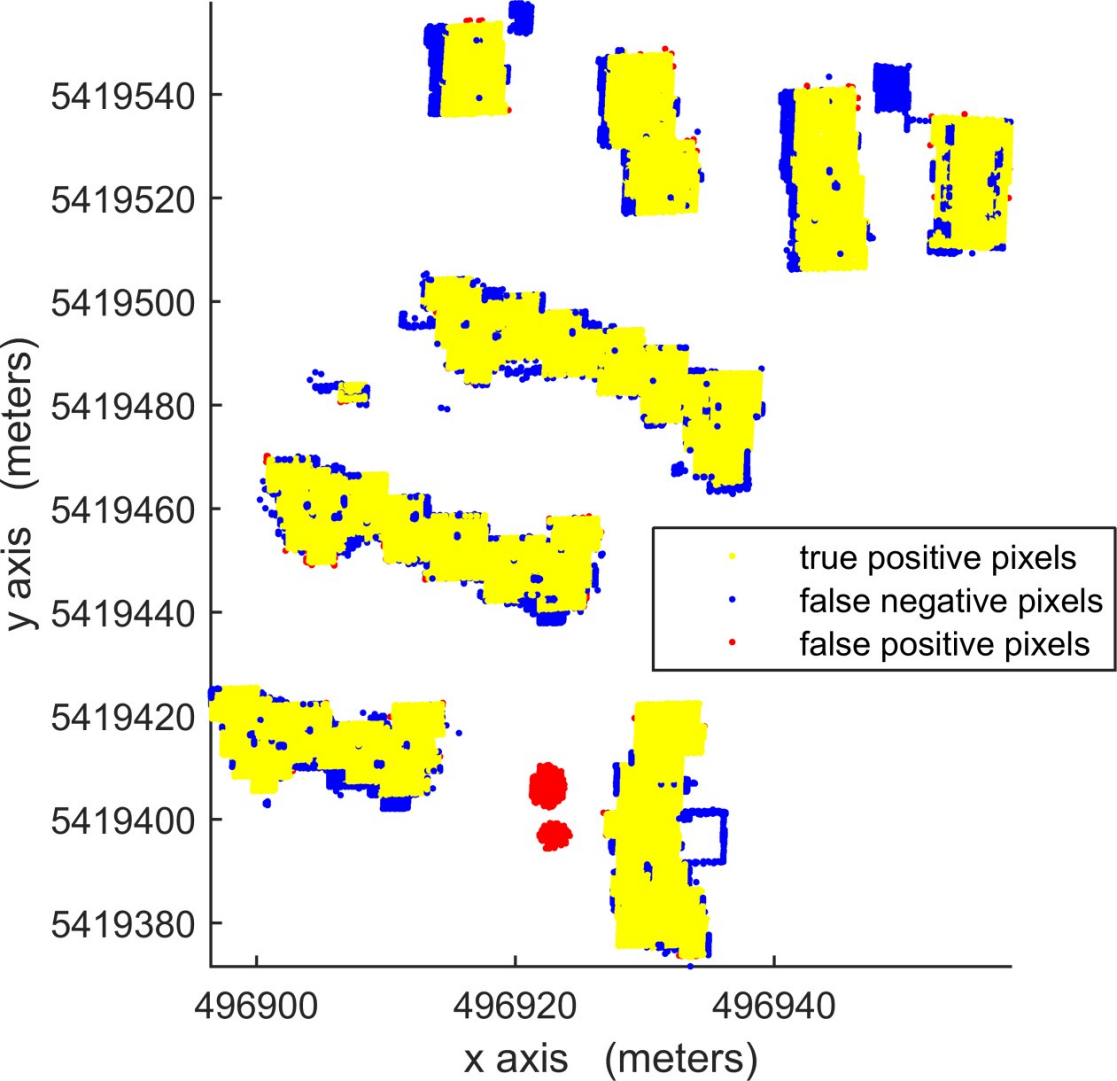
Top views of the building detection results and errors of the proposed VSBD algorithm for Area 2. with the vertical voxel resolution scheme of Eq (1).

**Fig 23:**
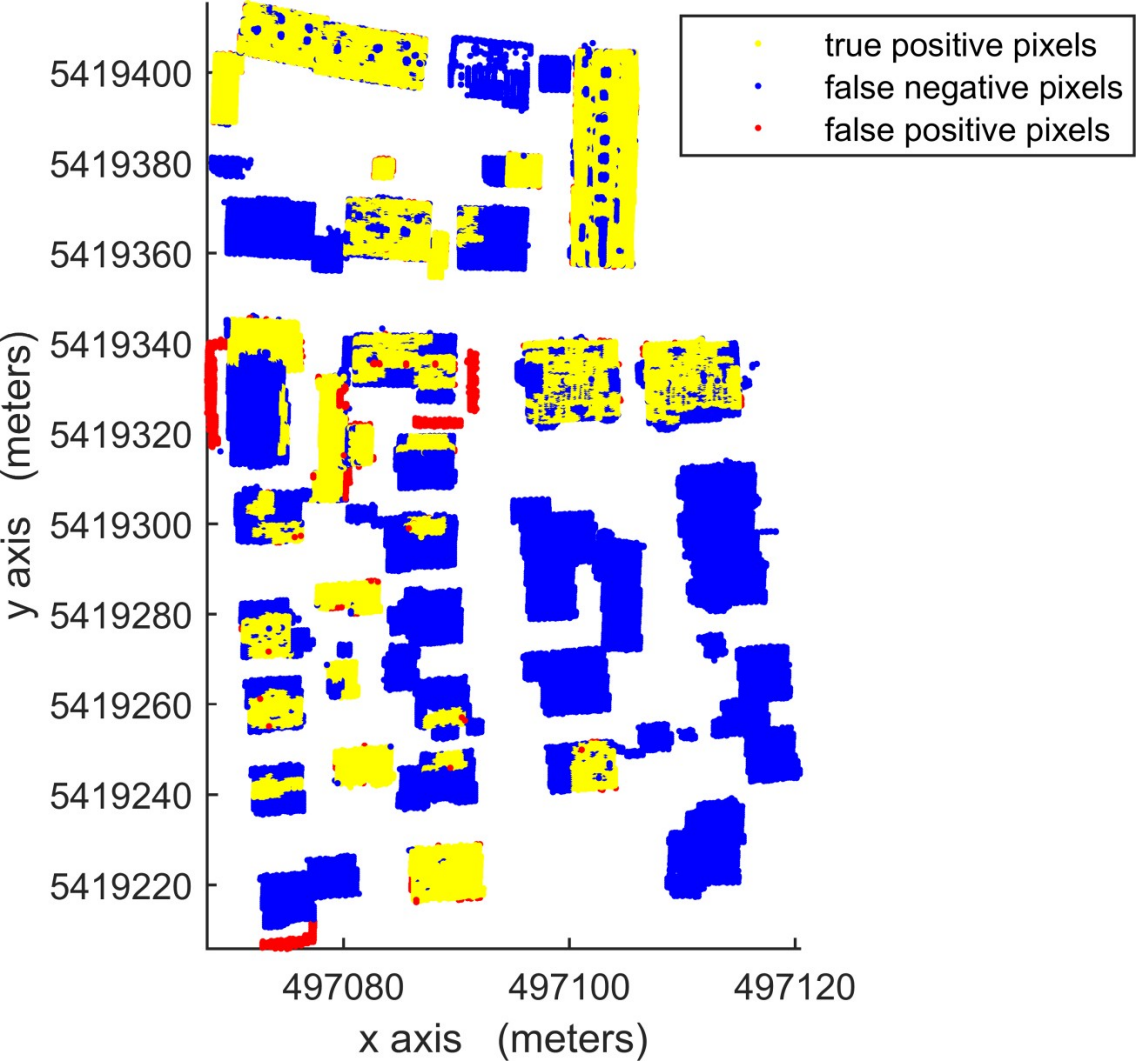
Top views of the building detection results and errors of the proposed VSBD algorithm for Area 3 with the vertical voxel resolution scheme of Eq (1).

Table 4 shows the average completeness, correctness and quality were 72.0%, 94.4% and 69.0%, respectively, for building detection. These indexes are obviously lower than those of the vertical resolution scheme of Δ*z* = Δ*x*. Moreover, in per-area mode, the building detection qualities of Areas 1, 2 and 3 were 76.1%, 86.7% and 44.2%, respectively. This indicates that the proposed VSBD algorithm with the vertical voxel resolution scheme of Eq (1) generates promising performance in Area 2 and has much worse performance in Areas 1 and 3 (see Figs 21, 22 and 23). This finding could be due to the following factors. Most buildings in Area 2 are flat-roofed and voxels corresponding to the same building roof can be grouped into a 3D-connected region even if vertical resolution is too high, and the 3D-connected region can be detected correctly based on its area, elevation difference and density characteristics (see Fig 22). However, Areas 1 and 3 contain many peak-roofed buildings and voxels corresponding to the same building roof may be grouped into multiple 3D-connected regions because the vertical resolution is too high, and the 3D-connected regions corresponding to the same building may be discarded according to their area or elevation difference characteristics (see Figs 21 and 23). The results are poor quality in Areas 1 and 3. Thus, a 0.1 m vertical resolution is too high and is not suitable for building detection. To summarize, the vertical resolution scheme of Δz = Δx is a more appropriate vertical resolution scheme for building detection.

### Comparative algorithm performance

To validate the performance of the proposed VSBD algorithm, its per-area accuracies for detected buildings were compared with ten of the best algorithms in the ISPRS link at http://www2.isprs.org/commissions/comm3/wg4/results/a1_detect.html, http://www2.isprs.org/commissions/comm3/wg4/results/a2_detect.html and http://www2.isprs.org/commissions/comm3/wg4/results/a3_detect.html (these three URLs correspond to the accuracies of Area 1, 2 and 3, respectively). The results are listed in Table 5.

**Table 5:** Per-area accuracies for detected buildings using the proposed VSBD algorithm and other algorithms.

Researchers	Area 1			Area 2			Area 3		
	Completeness (%)	Correctness(%)	Quality (%)	Completeness (%)	Correctness(%)	Quality (%)	Completeness (%)	Correctness(%)	Quality (%)
**Our**	90.6	94.5	86.0	93.8	**98.7**	**92.7**	*85*.*5*	*94*.*9*	*85*.*7*
**DLR**	91.9	95.4	88.0	94.3	97	**91.6**	93.7	95.5	89.7
**ZJU**	92.1	95.2	88.0	**94.4**	97.7	92.3	92	96.3	88.9
**LJU2**	**94.6**	*93*.*1*	88.4	95.1	*94*.*3*	89.9	**94.6**	95.6	**90.7**
**LJU1**	93.2	94.1	88.1	95.1	*94*.*3*	89.9	**94.4**	95.4	**90.3**
**CSU**	**93.6**	94.5	88.8	**94.6**	*95*.*4*	90.5	93.9	*94*.*7*	89.2
**HKP**	92.0	**97.4**	**89.8**	93	98.4	91.6	89.2	97.7	87.4
**HANC3**	90.8	94.5	86.2	91.4	96.4	88.4	91.6	96.7	88.8
**CAL2**	*87*.*7*	97.2	*85*.*6*	90.7	96.7	*88*	89.2	**97.7**	87.4
**MAR2**	*90*.*3*	*91*.*7*	*83*.*5*	*89*.*9*	97.8	88.1	*88*.*9*	96.2	*85*.*9*
**WHU_YD**	91.8	**98.6**	**90.6**	*87*.*3*	**99**	*86*.*5*	90.2	**98.1**	88.7

The two highest values per column are shown in bold and the two worst values are shown in italics.

As shown in Table 5, the detected building result of the proposed VSBD algorithm for Area 2 achieves the maximum quality metric in per-area mode whreeas that for Area 3 is the minimum quality metric. The main reason of the poor quality assessment for Area 3 is that buildings with very low LIDAR point density cannot be detected (see the brown rectangles in Fig 18). The 3D connectivity of buildings with very few returns is disrupted, leading to the misclassification in Area 3. Future studies should focus on recovering the 3D connectivity of buildings in the GVS by virtue of the related operation (e.g., dilation) of 3D mathematical morphology to improve the generalization of the proposed VSBD algorithm.

## Discussion and conclusions

A VSBD algorithm for airborne LIDAR data is proposed to detect building objects in urban scenes. The proposed VSBD algorithm first constructs a GVS model of airborne LIDAR data to comprehensively utilize the elevation and grayscale information. The constructed GVS is segmented into multiple 3D-connected regions by relying on the connectivity and grayscale similarity among voxels. The 3D-connected regions corresponding to the building roof and facade are detected sequentially using their characteristics. The ISPRS-WGIII/4 dataset with different building types was used to evaluate the performance of the proposed VSBD algorithm with manually selected parameters for each testing site and to compare the performance of the proposed VSBD algorithm with those of ten published algorithms. The per-area quantitative evaluation results indicate that (1) the average quality, completeness and correctness indexes are 88.1%, 90.0% and 96.0%, respectively, and (2) compared to other algorithms, the proposed VSBD algorithm achieves maximum quality in an environment with high-rising residential buildings surrounded by trees and high quality in inner city environments and purely residential areas with small detached houses. In general, the proposed VSBD algorithm is helpful to comprehensively utilize multiple returns to improve the accuracy of the building detection results and can be used to detect 3D buildings. However, the GVS in the proposed VSBD algorithm only fuses the elevation and intensity information, which makes it suitable only for distinguishing objects with different elevations or intensities. Future work will include assigning attributes from associated imagery to improve the classification of more complex scenes.
